# Development, structure, and mechanism of synthetic antibodies that target claudin and *Clostridium perfringens* enterotoxin complexes

**DOI:** 10.1016/j.jbc.2022.102357

**Published:** 2022-08-09

**Authors:** Benjamin J. Orlando, Pawel K. Dominik, Sourav Roy, Chinemerem P. Ogbu, Satchal K. Erramilli, Anthony A. Kossiakoff, Alex J. Vecchio

**Affiliations:** 1 Department of Biochemistry and Molecular Biology, Michigan State University, East Lansing, Michigan, USA; 2 Department of Biochemistry and Molecular Biology, University of Chicago, Chicago, Illinois, USA; 3 Department of Biochemistry, University of Nebraska-Lincoln, Lincoln, Nebraska, USA

**Keywords:** claudins, tight junctions, membrane proteins, cryogenic electron microscopy, synthetic antibody fragments, BLI, bio-layer interferometry, cCpE, CpE’s C-terminal domain, COP, CpE obstructing protein, CpE, *Clostridium perfringens* enterotoxin, DDM, n-dodecyl-β-D-maltopyranoside, ECS, extracellular segment, NHS-PEG4-biotin, N-hydroxysuccinimide polyethylene glycol biotin, SEC, size-exclusion chromatography, sFab, synthetic antigen-binding fragment, TM, transmembrane

## Abstract

Strains of *Clostridium perfringens* produce a two-domain enterotoxin (CpE) that afflicts humans and domesticated animals, causing prevalent gastrointestinal illnesses. CpE’s C-terminal domain (cCpE) binds cell surface receptors, followed by a restructuring of its N-terminal domain to form a membrane-penetrating *β*-barrel pore, which is toxic to epithelial cells of the gut. The claudin family of membrane proteins are known receptors for CpE and also control the architecture and function of cell-cell contacts (tight junctions) that create barriers to intercellular molecular transport. CpE binding and assembly disables claudin barrier function and induces cytotoxicity via *β*-pore formation, disrupting gut homeostasis; however, a structural basis of this process and strategies to inhibit the claudin–CpE interactions that trigger it are both lacking. Here, we used a synthetic antigen-binding fragment (sFab) library to discover two sFabs that bind claudin-4 and cCpE complexes. We established these sFabs’ mode of molecular recognition and binding properties and determined structures of each sFab bound to claudin-4–cCpE complexes using cryo-EM. The structures reveal that the sFabs bind a shared epitope, but conform distinctly, which explains their unique binding equilibria. Mutagenesis of antigen/sFab interfaces observed therein result in binding changes, validating the structures, and uncovering the sFab’s targeting mechanism. From these insights, we generated a model for CpE’s claudin-bound *β*-pore that predicted sFabs would not prevent cytotoxicity, which we then verified *in vivo*. Taken together, this work demonstrates the development and mechanism of claudin/cCpE-binding sFabs that provide a framework and strategy for obstructing claudin/CpE assembly to treat CpE-linked gastrointestinal diseases.

Tight junctions are molecular gatekeepers that regulate transport of small molecules through the paracellular spaces between adjoining cells in endothelia and epithelia. To accomplish this function, tight junctions possess integral membrane proteins that self-assemble to simultaneously span both intracellular and paracellular spaces (1, 2). Of the numerous membrane proteins at tight junctions, the 27-member family of claudins comprise the major structural and functional backbone of tight junctions, making them attractive targets to modulate tight junction barriers therapeutically (3). Evolution has successfully accomplished this feat. Type F strains of the pathogenic Gram-positive bacterium *Clostridium perfringens* produce an enterotoxin (CpE) that binds claudins to dissociate tight junctions during cytotoxicity in the gut (4, 5, 6). In domesticated animals, CpE causes necrotic enteritis, colitis, and diarrhea (7, 8). In humans, CpE causes enterotoxemia, which is the third most prevalent foodborne illness in the United States causing an estimated ∼$400 million annual economic burden and is the source of a further 4+ million food poisoning cases worldwide—some resulting in death (8, 9, 10, 11, 12). Unlike other human diseases caused by *C. perfringens* toxins, CpE-associated ailments are not directly preventable nor treatable, and no vaccine exists against this food poisoning type (13). Because spore-formed *C. perfringens* is heat-resistant, cooking does little to reduce its pathogenicity, as digested spores can produce CpE (14). Therefore, submolecular details into CpEs mechanisms of claudin binding and dissociating tight junction barriers are essential to elucidate CpE cytotoxicity and to develop therapeutic strategies for CpE-based diseases.

The C-terminal domain of CpE (cCpE) selectively targets claudins in the gut by recognizing a motif unique to these receptors, then binds them with low nanomolar affinities (15, 16). This claudin-bound CpE, that is, “small complex”, then oligomerizes and its N-terminal domain structurally rearranges to form a membrane-penetrating and cytotoxic *β*-barrel pore (17, 18). The process of *β*-pore formation disables claudin–claudin interactions vital to tight junction assembly and ultimately dissociates tight junctions causing paracellular leakage prior to CpE-induced cell death. Crystal structures of cCpE bound to receptor claudins have shed light on their interprotein interactions and have helped to inform structure-guided design of modified cCpEs used to detect or destroy cancer cells or to modulate the blood-brain barrier for drug delivery (16, 19, 20, 21, 22, 23, 24, 25, 26). Yet, a complete structural and mechanistic understanding of CpE dissociation of tight junctions and the process of cytotoxic *β*-pore formation remains elusive.

We intended to elucidate how CpE binds claudins and dissociates tight junctions by determining X-ray crystal structures of enterotoxins CpE or cCpE in complex with claudins but found crystallization to be a bottleneck. For most claudins, crystals did not form at all, while for those that formed crystals, it required screening and optimizing hundreds over ∼1 year to determine structures resolved to 3 to 4 Å (1,6, 24). Using a phage display, library-encoding synthetic antigen-binding fragments (sFabs), we sought to discover molecules that target and bind complexes between enterotoxins and human claudin-4 (claudin-4). Our goal was to use sFabs to chaperone crystallization, improve initial diffraction, and increase structural throughput of this and other claudin–enterotoxin complexes (27, 28). Through this approach, we surmised additional sFabs could discover that obstruct complex formation altogether and be useful in therapeutic development. During this process, three sFabs were discovered, which we termed CpE Obstructing Proteins (COPs). Preliminary characterization of COPs revealed that COP-2 and COP-3 had properties amenable for structure determination of the claudin-4–cCpE complex. Ultimately, however, we determined a structure of this complex using a traditional crystallography workflow (16). But because sFabs have recently been shown effective for determining structures of small membrane proteins and complexes by cryo-EM, we used COP-2, COP-3, and cryo-EM to progress a novel workflow for higher throughput elucidation of claudin/enterotoxin structures (29, 30, 31).

Here, we qualitatively and quantitatively characterize COP-2 and COP-3 binding to claudin-4, cCpE, and CpE individually, and to claudin-4–enterotoxin complexes using biochemical and biophysical techniques. We also use cryo-EM at 200 kV to determine 4 to 7 Å structures of each 50 kDa COP bound to ∼35 kDa claudin-4–cCpE complexes in ∼1 month and employ these structures to create models of the cytotoxic CpE *β*-pore. Our findings reveal the structural basis and COP-specific mechanisms of COP targeting of claudin-4–cCpE complexes. This research independently validates claudin-4/cCpE structures determined by X-ray crystallography and provides a structural framework for preventing CpE-mediated cytotoxicity by obstructing toxin/receptor binding. Moreover, it advances development of technologies and establishes a general approach for determining structures of other claudin–enterotoxin complexes at moderate resolutions using accessible cryo-EM instrumentation that can readily be expanded to higher resolutions with 300 kV microscopes. Further, it demonstrates the antigenicity of CpE and cCpE enterotoxins and their claudin-bound complexes, which through intensified sFab development could generate novel sFabs useful for modulating tight junction barriers or as therapeutics for preventing or treating CpE-based illnesses in humans and domesticated animals.

## Results

### Development of COPs

Claudin-4 solubilized in n-dodecyl-*β*-D-maltopyranoside (DDM) and bound to cCpE was used as input for phage display selection using a large and diverse library of sFabs based on a humanized Fab scaffold. The sFab library has varied sequences that are biased for serine and tyrosine in the complementarity-determining regions (CDRs) of their light (L) and heavy (H) chains within variable domains (32, 33). After several rounds of selection to increase stringency, two sFabs, termed COP-2 and COP-3, were further developed and validated by ELISA to bind to claudin-4/cCpE. Both COPs were sequenced and isolated to characterize their binding further. Sequence alignments of COP-2 and COP-3 reveal that COP-2 and COP-3 share 98.2 and 93.3% sequence identity in their L and H chains, respectively; and that COP sequences diverge the greatest in CDR-L3, CDR-H1, and CDR-H3 (Supporting Information (SI) Fig. S1). These residue divergences may direct COP-specific recognition of claudin-4–cCpE complexes.

### Biochemical characterization of COPs

**Figure 1:**
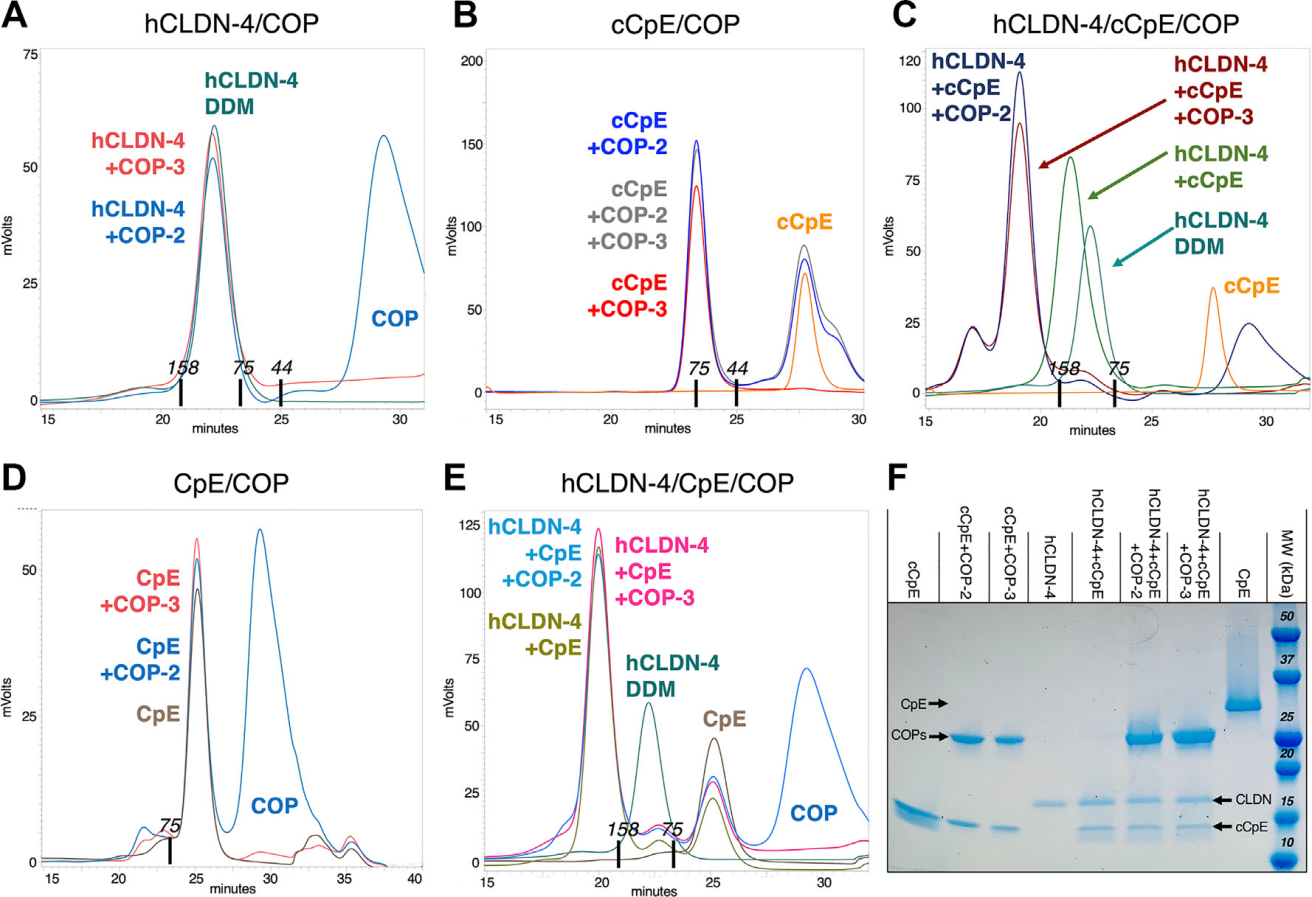
**Biochemical characterization of COP molecular recognition**. Human claudin-4 (hCLDN-4) in DDM, cCpE, CpE, and COP-2 or COP-3 were incubated together and then injected onto a SEC column equilibrated in DDM and monitoring using 280 nm absorbance. The SEC traces depict the following: COP-2 (*blue*) and/or COP-3 (*red*) binding to (*A*) claudin-4 alone (*green*), (*B*) cCpE alone (*orange*), (*C*) claudin-4–cCpE complexes (*green*), (*D*) CpE alone (*brown*), and (*E*) claudin-4–CpE complexes (*brown green*). SEC elution times of MW standards are shown near X axes. (*F*), SEC peak fractions from (*A*-*E*) were pooled and subjected to SDS-PAGE. cCpE, CpE’s C-terminal domain; COP, CpE obstructing protein; DDM, n-dodecyl-*β*-D-maltopyranoside; SEC, size-exclusion chromatography.

To obtain more detailed insights into COP recognition, we determined which molecule COPs bind and if they use unique or common epitopes. After expressing and purifying claudin-4, cCpE, CpE, and COPs, increases in molecular masses as assessed by decreases in peak retention times with size-exclusion chromatography (SEC) was used to qualitatively characterize COP-2 and COP-3 binding modes. After incubating COP-2 and COP-3 with claudin-4 and cCpE alone, we found that COPs did not bind claudin-4 but did form larger complexes with cCpE (Fig. 1, *A* and *B*). Incubating claudin-4, cCpE, and COPs together showed that cCpE binds claudin-4 and that both COPs bound claudin-4–cCpE complexes (Fig. 1*C*). No mass increases were observed for COPs incubated with CpE (Fig. 1*D*). Lastly, incubating claudin-4, CpE, and COPs together showed that while CpE binds claudin-4 and forms a “small complex”, the COPs do not bind “small complexes” (Fig. 1*E*). To verify complex formation, peaks from SEC were pooled and subjected to SDS-PAGE, which showed the presence of individual proteins from associated complexes (Fig. 1*F*). To determine if COPs share a binding epitope, we incubated COP-2 and COP-3 together with cCpE. If COPs bound distinct epitopes, a molecular mass shift greater than the individual cCpE–COP complex would result. SEC revealed no additive mass shift with both COPs present (Fig. 1*B*). These biochemical results suggest the specific molecular recognition of COPs and that COPs share binding epitopes.

### Biophysical characterization of COPs

After qualitatively establishing COP binding, we quantitated the affinities and kinetics of COP interactions with claudin-4 and enterotoxins. We determined the second-order association rate constant (k_on_), first-order dissociation rate constant (k_off_), and equilibrium dissociation constant (K_D_) of these interactions using bio-layer interferometry (BLI) (Table 1; SI Fig. S2). BLI measurements were made using preformed claudin-4–enterotoxin complexes or cCpE alone in DDM, replicating the conditions of the phage display selections. For COP-2 and COP-3 binding to claudin-4–cCpE complexes, we measured K_Ds_ of 52.3 and 98.4 nM, respectively. Comparing the binding rates revealed that COP-3 had 1.9- and 3.5-fold faster k_on_ and k_off_ rates than COP-2. The K_Ds_ of COP-2 and COP-3 to cCpE were 67.6 and 137.6 nM, respectively. Like COP-3 binding to claudin-4–cCpE complexes, the k_on_ and k_off_ rates were 1.4- and 2.1-fold faster than COP-2. Finally, we measured K_Ds_ of 7.8 and 9.1 μM for COP-2 and COP-3 binding to claudin-4/CpE “small complexes”, respectively. The K_D_ values represent 149.1- and 92.0-fold decreases in COP-2 and COP-3 affinity for CpE compared to cCpE. Kinetic differences in k_on_ and k_off_ of COP binding to cCpE are visible in the BLI sensorgrams (SI Fig. S2). These results agree with biochemical assessment and reveal biophysical parameters unique to each COP that may influence recognition and binding to claudin-4–cCpE complexes.

**Table 1:** Affinities and kinetics of COP binding to claudin-4/cCpE and enterotoxins

COP	Complex/Protein	k_on_ (1/Ms)	k_off_ (1/s)	K_D_ (nM)
COP-2	claudin-4/cCpE	1.5x10^4^ ± 0.1x10^4^	7.8x10^−4^ ± 0.6x10^−4^	52.3 ± 3.0
	cCpE	7.2x10^3^ ± 0.6x10^3^	4.8x10^−4^ ± 0.4x10^−4^	67.6 ± 11.7
	claudin-4/CpE	1.5x10^2^ ± 0.6x10^2^	1.2x10^−3^ ± 0.6x10^−3^	7800 ± 750
COP-3	claudin-4/cCpE	2.9x10^4^ ± 0.7x10^4^	2.7x10^−3^ ± 0.5x10^−3^	98.4 ± 11.0
	cCpE	1.0x10^4^ ± 0.1x10^4^	1.0x10^−3^ ± 0.1x10^−3^	137.6 ± 6.2
	claudin-4/CpE	1.2x10^3^ ± 0.2x10^3^	10.8x10^−2^ ± 0.7x10^−2^	9050 ± 1300

### Cryo-EM structures of claudin-4–cCpE–COP complexes

**Figure 2:**
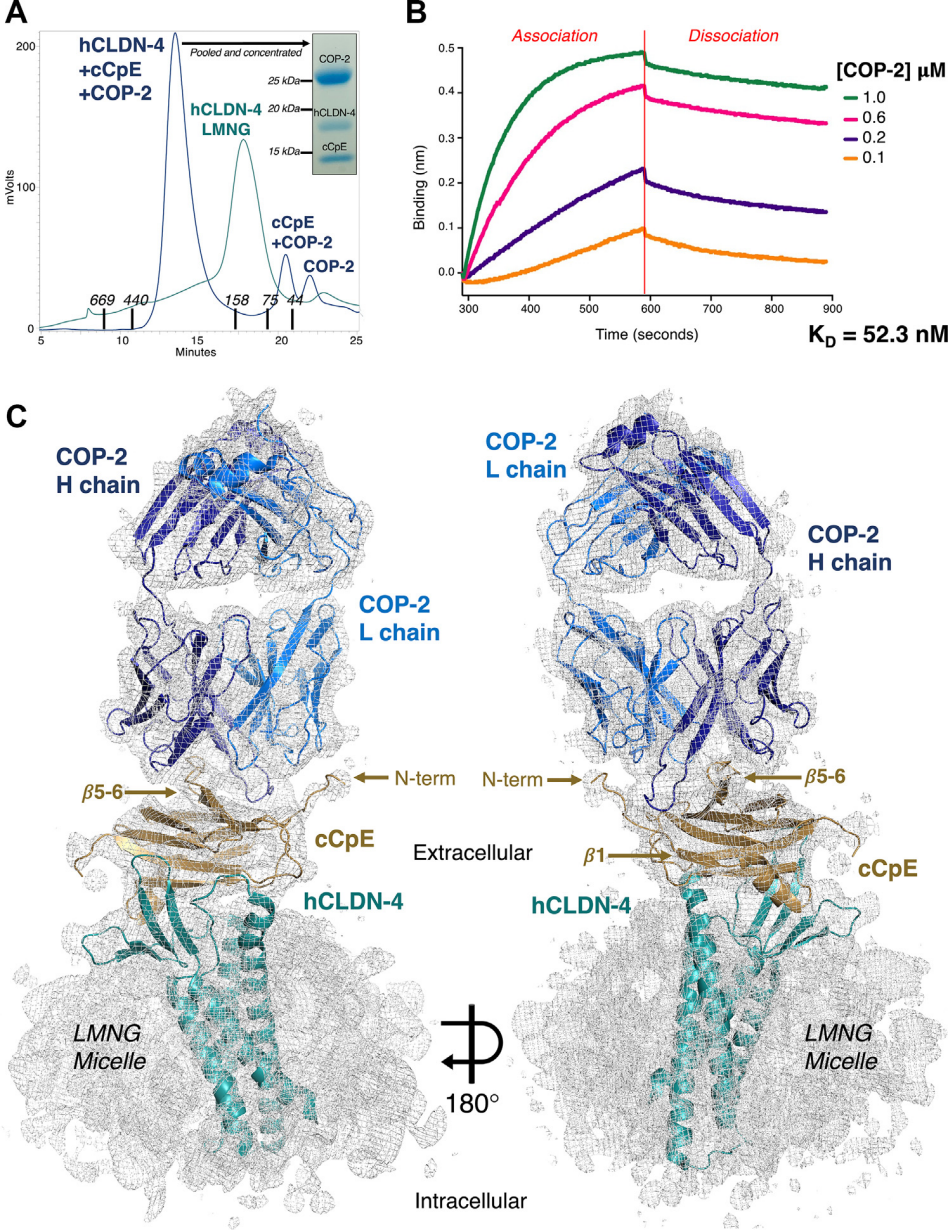
**Structure and function of COP-2 binding to claudin-4–cCpE complexes.***A*, SEC purification of human claudin-4 (hCLDN-4)–cCpE–COP-2 complexes in LMNG. SEC elution times of MW standards are shown by X axis. *B*, binding of COP-2 to claudin-4/cCpE using BLI. *C*, cryo-EM structure of COP-2 (*blue*) bound to cCpE (*copper*) and hCLDN-4 (*teal*). The cryo-EM map (*gray mesh*) is contoured to 4.0 σ. cCpE, CpE’s C-terminal domain; COP, CpE obstructing protein; SEC, size-exclusion chromatography; BLI, bio-layer interferometry.

Having confirmed that COP-2 binds claudin-4–cCpE complexes using SEC (Fig. 2*A*) and BLI (Fig. 2*B*), we next delineated the structural basis and molecular mechanism of COP-2 targeting by determining its structure by cryo-EM. Cryo-EM screening of claudin-4–cCpE–COP-2 complexes that were SEC purified, pooled, and concentrated to 8.0 mg/ml in various detergents showed that those solubilized in 2,2-didecylpropane-1,3-bis-*β*-D-maltopyranoside (LMNG) were amenable to structure determination. This was confirmed by 2D classifications and *ab initio* 3D reconstructions, which revealed a stacked and linear arrangement of the three proteins, a canonical two-lobed sFab, and flexibility in COP-2’s constant domain (SI Fig. S3). Due to this flexibility, we used a data processing strategy that masked claudin-4, cCpE, and the variable domains of COP-2 to resolve and focus understanding on the interactions directing COP-2 recognition. Using this strategy, a cryo-EM map that was resolved to 6.9 Å was generated (SI Table S1). The map resolution was sufficient to reveal the claudin-4–cCpE–COP-2 complex, secondary structural elements including claudin-4’s four transmembrane helices (TM), and some bulky side chains (SI Fig. S4*A*). This cryo-EM map was used to build, refine, and determine a structure for the claudin-4–cCpE–COP-2 complex. SI Table S1 shows data processing, refinement, and model-to-map fit statistics, with further details given in Experimental procedures.

The cryo-EM map resolution was insufficient to place side chains confidently, so we cannot verify interactions between claudin-4 and cCpE that define the “cCpE-binding motif” (16). Overall, however, the claudin-4/cCpE portion of this complex from cryo-EM superimposed well onto the crystal structure (SI Fig. S5*A*). We measured RMSDs in C⍺ positions of 2.0 and 1.4 Å between the cores of claudin-4 and cCpE and 1.7 Å in overall secondary structures between the cryo-EM and crystal structures of the claudin-4–cCpE complex, indicating no major conformational changes occur upon COP-2 binding. Generally, the claudin-4 extracellular segments (ECSs) are in similar conformations and thus may interact with cCpE similarly in the cryo-EM and crystal structures (SI Fig. S5*A*). Density corresponding to a loop within ECS2 that contains the NPLVA^153^ motif shows that the motif accesses a groove on the surface of cCpE. This interaction is known to impart high-affinity cCpE binding to claudins, and COP-2 appears to not significantly alter its structure (16).

The cryo-EM structure of the claudin-4–cCpE–COP-2 complex reveals the basis of COP-2 binding. The canonical binding of cCpE to claudin-4 exposes cCpE’s top half to COP-2 binding by providing an antigenic surface (Fig. 2*C*). COP-2 binding to cCpE alters the conformations of cCpE’s N-terminus, *β*-strands *β*5 and *β*6, and the loop connecting them, when compared to the crystal structure (SI Fig. S5*A*). COP-2’s L chain sits atop a depression on the exterior of cCpE formed between the N-terminus and strands *β*5 and *β*6 (Fig. 2*C*). Chain L’s CDR-L1, CDR-L2, and CDR-L3 conform to the surface of cCpE and potential side chains involved in these interactions can be visualized although not placed confidently (Fig. 4, *A* and *B*). COP-2’s chain H accesses the same surface depression as chain L but also flanks the opposite side of strands *β*5 and *β*6—CDR-H3 shares an epitope with chain L while CDR-H1 and CDR-H2 reside on the other (Fig. 2*C* and Fig. 4*C*). CDR-H3 splays outward to deeply penetrate its surface groove, conforming to the cCpE surface (Fig. 4*D*). Based on the interactions projected by the structure, we hypothesized that residues comprising Lys197 to Leu202 and Asn267 to Gln276 in cCpE could influence COP-2 binding.

**Figure 3:**
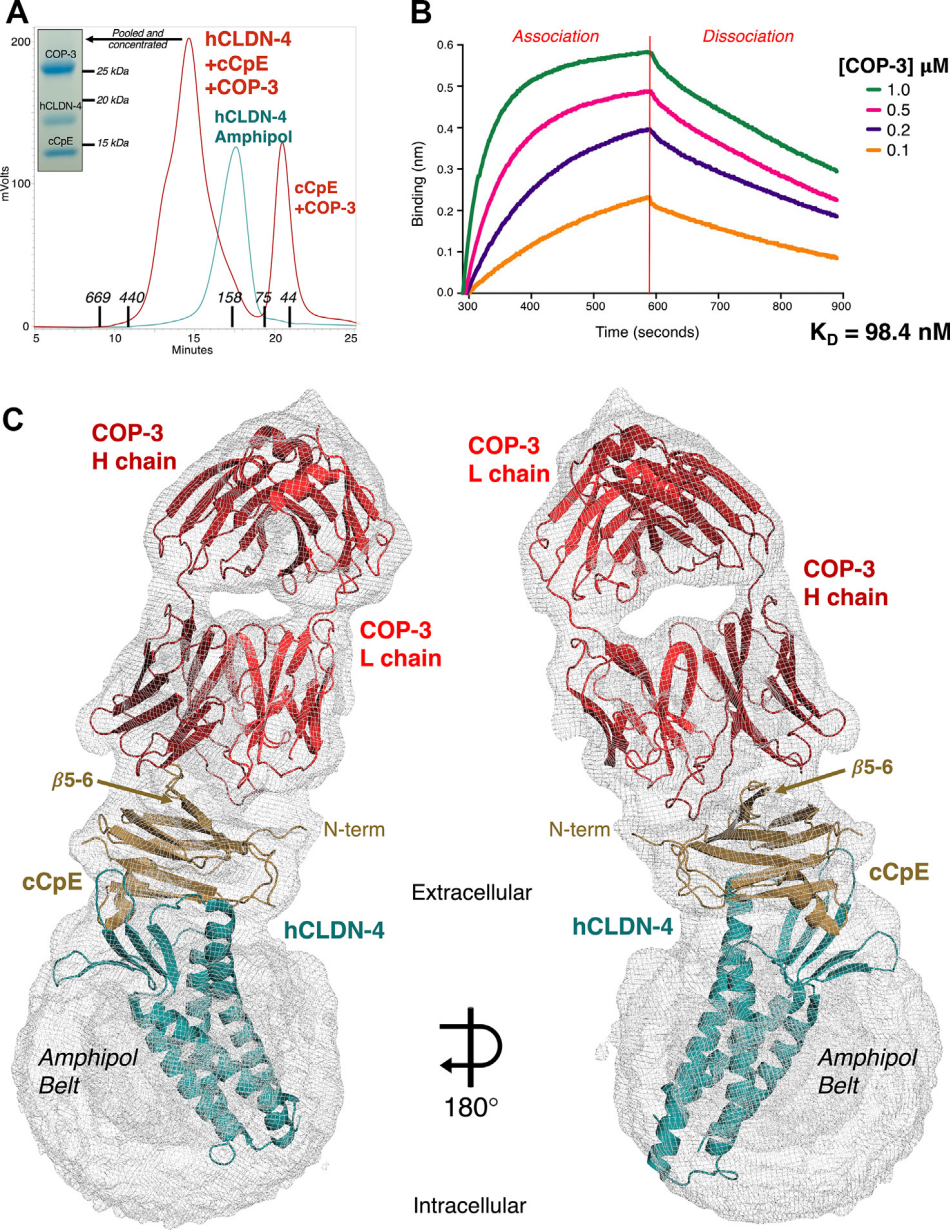
**Structure and function of COP-3 binding to claudin-4–cCpE complexes.***A*, SEC purification of hCLDN-4–cCpE–COP-3 complexes in amphipol. SEC MW standard elution times are shown by X axis. *B*, binding of COP-3 to claudin-4/cCpE using BLI. *C*, cryo-EM structure of COP-3 (*red*) bound to cCpE (*copper*) and hCLDN-4 (*teal*). The cryo-EM map (*gray mesh*) is contoured to 5.0 σ. cCpE, CpE’s C-terminal domain; COP, CpE obstructing protein; SEC, size-exclusion chromatography; BLI, bio-layer interferometry.

After verifying that COP-3 binds claudin-4–cCpE complexes using SEC (Fig. 3*A*) and BLI (Fig. 3*B*), we next determined a structure for COP-3 in complex with claudin-4/cCpE by cryo-EM to contrast its molecular mechanism of targeting with COP-2. Claudin-4–cCpE–COP-3 complexes in various membrane mimetics were SEC purified, pooled, and concentrated to 6.0 mg/ml for cryo-EM. Those complexes solubilized in amphipol were superior to LMNG and DDM for cryo-EM based on 2D class averages. The 2D and 3D classifications of the COP-3 complex showed a single complex with each protein stacked in a linear arrangement and had features corresponding to secondary structural elements and both lobes of COP-3 (SI Fig. S6). The 3D reconstructions showed that the constant domains of COP-3 are less dynamic than those of COP-2, but also that claudin-4’s TM region was less resolved in amphipol. We used a data processing strategy that masked COP-3 to best resolve the interactions directing its recognition of claudin-4/cCpE, which generated map (focused) that was resolved to 3.8 Å and showed structural features including density for bulky side chains (SI Table S1). However, this focused map lacked definition for cCpE and claudin-4, and model-to-map fit proved difficult. Therefore, data were processed with no mask, which produced a map (whole) that was resolved to 5.0 Å. The whole map resolved the claudin-4–cCpE–COP-3 complex, including secondary structural elements like claudin-4’s TMs, ⍺-helices and *β*-strands in cCpE, and the conformations of COP-3 CDRs (SI Fig. S4*B*). Initial model building and refinement employed both maps, but the final model was refined against the whole map, resulting in the cryo-EM structure for the claudin-4–cCpE–COP-3 complex. SI Table S1 shows data processing, refinement, and model-to-map fit statistics, with further details given in Experimental procedures.

**Figure 4:**
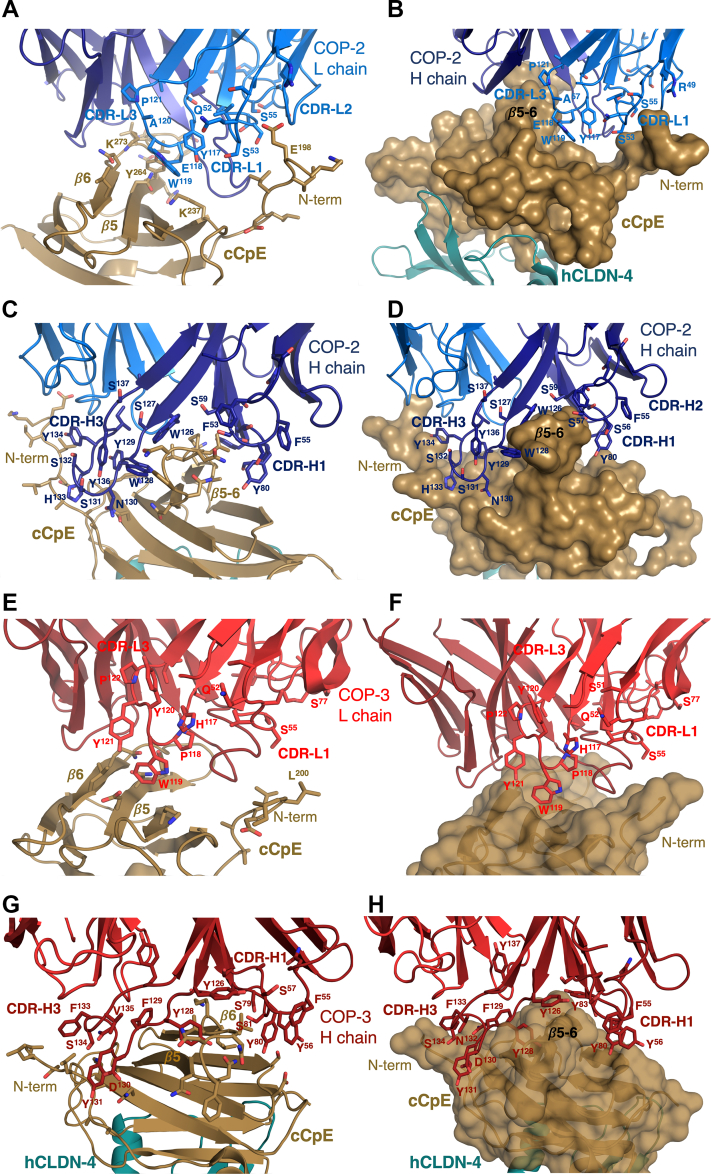
**COP recognition of cCpE epitopes.** Potential interactions between COP-2 (*blue*) and cCpE (*copper*) for the following: (*A* and *B*) chain L (*light blue*) and (*C* and *D*) chain H (*dark blue*). COP-2 and cCpE are both represented as cartoons (*A* and *C*) or COP-2 as a cartoon and cCpE as a surface (*B* and *D*). Potential interactions between COP-3 (*red*) and cCpE (*copper*) for the following: (*E* and *F*) chain L (*red*) and (*G* and *H*) chain H (*maroon*). COP-3 and cCpE are both represented as cartoons (*E* and *G*) or COP-3 as a cartoon and cCpE as a surface (*F* and *H*). cCpE, CpE’s C-terminal domain; COP, CpE obstructing protein.

The cryo-EM structure of the claudin-4–cCpE–COP-3 complex reveals COP-3’s mode of binding. Compared to the claudin-4/cCpE crystal structure, the equivalent portion from the COP-3 cryo-EM structure superimposes well and indicates that COP-3 does not induce large conformational changes or affect normal claudin-4–cCpE interactions (SI Fig. S5*B*). We measured RMSDs in C⍺ positions of 2.2 and 1.2 Å between the cores of claudin-4 and cCpE and 2.1 Å between overall secondary structures in the claudin-4–cCpE complex when comparing cryo-EM and X-ray structures. Like COP-2, COP-3 accesses cCpE’s surface opposite to where claudin-4 binds due their canonical interactions (Fig. 3*C*). COP-3 binding to cCpE alters cCpE’s N-terminus, *β*5 and *β*6, and the loop connecting them, when compared to the crystal structure (SI Fig. S5*B*). The L chain of COP-3 binds between cCpE’s N-terminus and *β*5 and *β*6 using CDR-L1 and CDR-L3 (Fig. 4*E*). The CDR-L3 loop conforms to the surface of this region, potentially using aromatic side chains to drive shape complementarity (Fig. 4*F*). COP-3’s H chain flanks both sides of *β*5 and *β*6 of cCpE with CDR-H3 sharing an epitope with chain L, while CDR-H1 and CDR-H2 occupy the other side (Fig. 3*C* and Fig. 4*G*). COP-3’s CDR-H3 conforms to and deeply accesses a surface groove between this region and the N-terminus (Fig. 4*H*). Based on the interactions approximated by the structure, we hypothesized that residues comprising Glu198 to Leu202 and Asn269 to Gln276 in cCpE may guide COP-3 binding.

### Comparison of COP structures

Because structure resolution was limiting, we used computational analyses to estimate the COP-specific residues used for cCpE recognition. For this, we input the structures into PDBePISA to determine the properties of the protein interfaces and then compared areas determined by PDBePISA to preside over cCpE/COP binding with COP primary sequences (SI Fig. S1) (34). For COP-2, we found that CDR-L3 residues Tyr117 to Ala120, CDR-H1 residue Ser56, and CDR-H3 residues Tyr125 to Ser137 were unique. For COP-3, we found that CDR-L3 residues His117 to Tyr121, CDR-H1 residue Tyr56, and CDR-H3 residues Gly125 to Tyr137 were unique. When compared to a generic sFab, COPs contain many aromatic side chains and are enriched in serine and tyrosine in their CDRs. This analysis exposed potential COP amino acid determinants for recognition and binding to claudin-4/cCpE.

**Figure 5:**
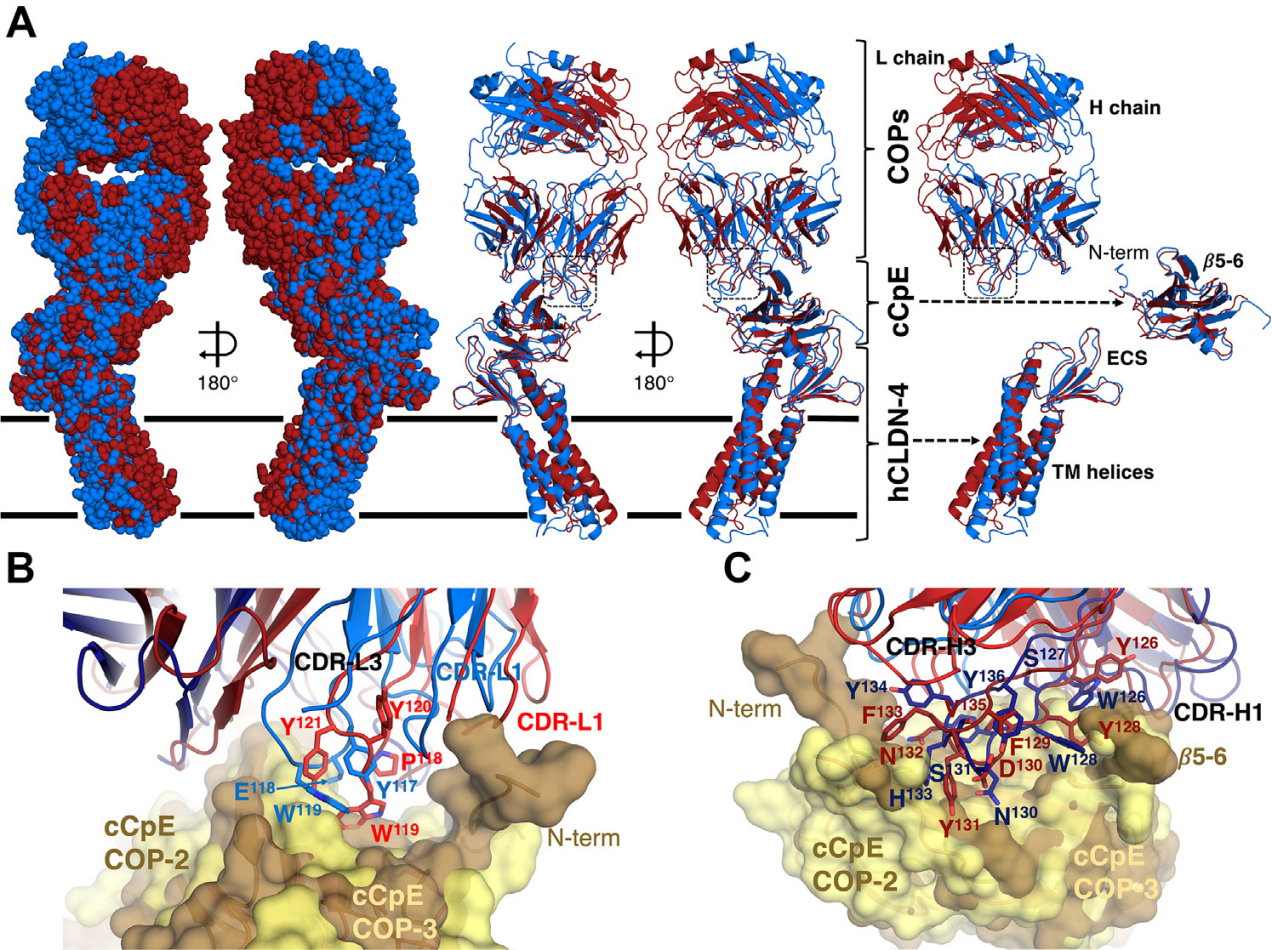
**Comparison of COP-2– and COP-3–bound claudin-4–cCpE complexes.** Structures were overlaid using Chimera (45). *A*, overlay of COP-2–bound (*blue*) and COP-3–bound (*maroon*) human claudin-4 (hCLDN-4)–cCpE complexes depicted as a surface (left) or cartoon (middle). Model membrane borders are shown as *black lines*. Overlays of each protein component were made by removing the other two components for ease of visualization and comparison (left). *B*, overlay of COP-2 L chain (*blue*) bound to cCpE (*copper*) and COP-3 L chain (*red*) bound to cCpE (*yellow*). COPs are represented as cartoons while cCpE is shown as a semitransparent surface. *C*, overlay of COP-2 H chain (*dark blue*) bound to cCpE (*copper*) and COP-3 H chain (*maroon*) bound to cCpE (*yellow*). Proteins are shown as in (*B*). cCpE, CpE’s C-terminal domain; COP, CpE obstructing protein.

To contrast COP binding modes and to explain their varied biophysical binding equilibria, we overlaid the two cryo-EM structures (Fig. 5). The overlays revealed that 1) the cCpE poses when bound to claudin-4 are similar but structural perturbations exist in cCpE due to COP-induced changes; 2) the claudin-4/cCpE portions exhibit minor structural differences between complexes but the claudin-4 TMs are oriented differently in membrane mimetics; and 3) the COPs have similar secondary structural elements, but their tertiary structures and CDRs vary in conformations (Fig. 5*A*). Focusing on regions with the largest observable differences, we found that the L and H chains of each COP conform to cCpE uniquely. For COP-2 chain L, CDR-L1 and CDR-L3 reside within the surface groove formed between the N-terminus and *β*5-*β*6 of cCpE while CDR-L2 lies external (Fig. 5*B*). For COP-3 chain L, the surface groove is depressed due to less N-terminal length, so while CDR-L1 and CDR-L3 reside in the same groove, CDR-L1 appears to interact less with cCpE. The conformation of CDR-H3 may force this CDR-L1 change in COP-3. With PDBePISA, we calculated interface surface areas between the L chain of COPs and cCpE and found that COP-2’s area was ∼36% larger. In both COP’s chain H, the CDR-H1 and CDR-H2 bind on one side of cCpE’s *β*5-*β*6 element while CDR-H3 flanks the other (Fig. 5*C*). While CDR-H1 and CDR-H2 have similar conformations and sequence alignments show that residue conservation is high, CDR-H3 conformations appear to vary and residue conservation is low (SI Fig. S1). Generally, the three CDR conformations overlay well between both COPs, which is reflected by cCpE/H chain interface surface area differences of only ∼5%. For both COPs, ∼80% of the total cCpE/COP interface area resides in chain H. Overall, COP-2 appears to use chain L to a higher degree than COP-3, while both COPs use chain H similarly and dominantly to bind cCpE. Moreover, the surface structure of cCpE appears to be altered more by COP-2 than COP-3, indicating that COP-specific interactions may uniquely mold cCpE’s surface due to sequence diversity (Fig. 5, *B* and *C*). We next determined if these structural features could explain the differences in binding equilibria between COP-2 and COP-3 found *in vitro*.

### Quantification of COP binding to mutant cCpEs

To test our structures and pinpoint the amino acid determinants of COP binding, we mutated regions of cCpE where we observed potential interactions with COPs and quantified binding with BLI. Preformed claudin-4–cCpE^mutant^ complexes were used to mimic the sFab selection experiment. First, we tested all mutants and found that no cCpE^mutant^ affected association with claudin-4, binding with K_D’s_ of ∼3 nM, similar to WT cCpE (SI Fig. S2*G*) (16, 35). We then tested COP binding to claudin-4/cCpE^mutant^ and compared to claudin-4/cCpE^WT^ (Table 2). BLI showed that mutant cCpE^1^, which has a truncated N-terminus, had decreased affinity for COPs. Mutant cCpE^2^ increased COP affinities, this mutation lies at the end of the N-terminus at the start of globular cCpE. Mutant cCpE^2^ with a Leu202Ala mutation, cCpE^2L^, showed binding to both COPs at near cCpE^WT^ levels but indicated a loss of affinity compared to cCpE^2^ due to the alanine side chain. Kinetics reveal that COPs bind claudin-4/cCpE^2L^ at different rates compared to claudin-4/cCpE^2^. The mutant cCpE^3^ in the loop connecting *β*5 to *β*6 bound with WT affinity to COP-2 but for COP-3 had 12-fold lower affinity. Both COPs exhibited substantial losses in affinity to mutant cCpE^4^, which alters residue lengths in the *β*5 to *β*6 loop and *β*6. COP-2 affinity decreased ∼270-fold while COP-3 decreased ∼40-fold. Unlike the other mutants, cCpE^4^ affected both kinetic rates, indicating that cCpE^4^ perturbs normal COP binding. To put these results in a structural context, we modeled mutant cCpEs based on our cryo-EM structures with cCpE^WT^ (SI Fig. S7). The models provide structural bases for COP binding to mutant cCpEs that explain our biophysical measurements. Overall, each set of cCpE mutants, chosen based on their potential for side chain interactions with COPs, altered COP binding in different ways, providing *in vitro* validation of our structures.

**Table 2:** Affinities and kinetics of COP binding to claudin-4/cCpE^mutant^ complexes

COP	Claudin-4/cCpE^mutant^	k_on_ (1/Ms)	k_off_ (1/s)	K_D_ (nM)
COP-2	CCpE^WT^	1.5x10^4^ ± 0.1x10^4^	7.8x10^−4^ ± 0.6x10^−4^	52.3 ± 3.0
	cCpE^1^	7.7x10^3^ ± 0.5x10^3^	1.2x10^−3^ ± 0.1x10^−3^	161.3 ± 0.6
	cCpE^2^	3.9x10^4^ ± 0.5x10^4^	3.3x10^−4^ ± 0.4x10^−4^	8.6 ± 0.1
	cCpE^2L^	1.7x10^4^ ± 0.1x10^4^	1.0x10^−3^ ± 0.1x10^−3^	57.2 ± 3.7
	cCpE^3^	1.3x10^4^ ± 0.1x10^4^	9.4x10^−4^ ± 0.1x10^−4^	64.5 ± 4.3
	cCpE^4^	2.9x10^2^ ± 0.1x10^2^	4.2x10^−3^ ± 0.1x10^−3^	14,200 ± 4970
COP-3	CCpE^WT^	2.9x10^4^ ± 0.7x10^4^	2.7x10^−3^ ± 0.5x10^−3^	98.4 ± 11.0
	cCpE^1^	1.9x10^4^ ± 0.2x10^4^	3.9x10^−3^ ± 0.6x10^−3^	200.2 ± 5.5
	cCpE^2^	11.0x10^4^ ± 2.2x10^4^	3.0x10^−3^ ± 0.1x10^−3^	24.6 ± 4.7
	cCpE^2L^	6.3x10^4^ ± 0.2x10^4^	5.3x10^−3^ ± 0.1x10^−3^	84.4 ± 4.8
	cCpE^3^	3.0x10^4^ ± 0.3x10^4^	3.9x10^−2^ ± 1.1x10^−2^	1225 ± 144
	cCpE^4^	2.7x10^3^ ± 0.2x10^3^	9.4x10^−3^ ± 0.1x10^−3^	4010 ± 687

### Model of cytotoxic claudin-bound CpE pore

**Figure 6:**
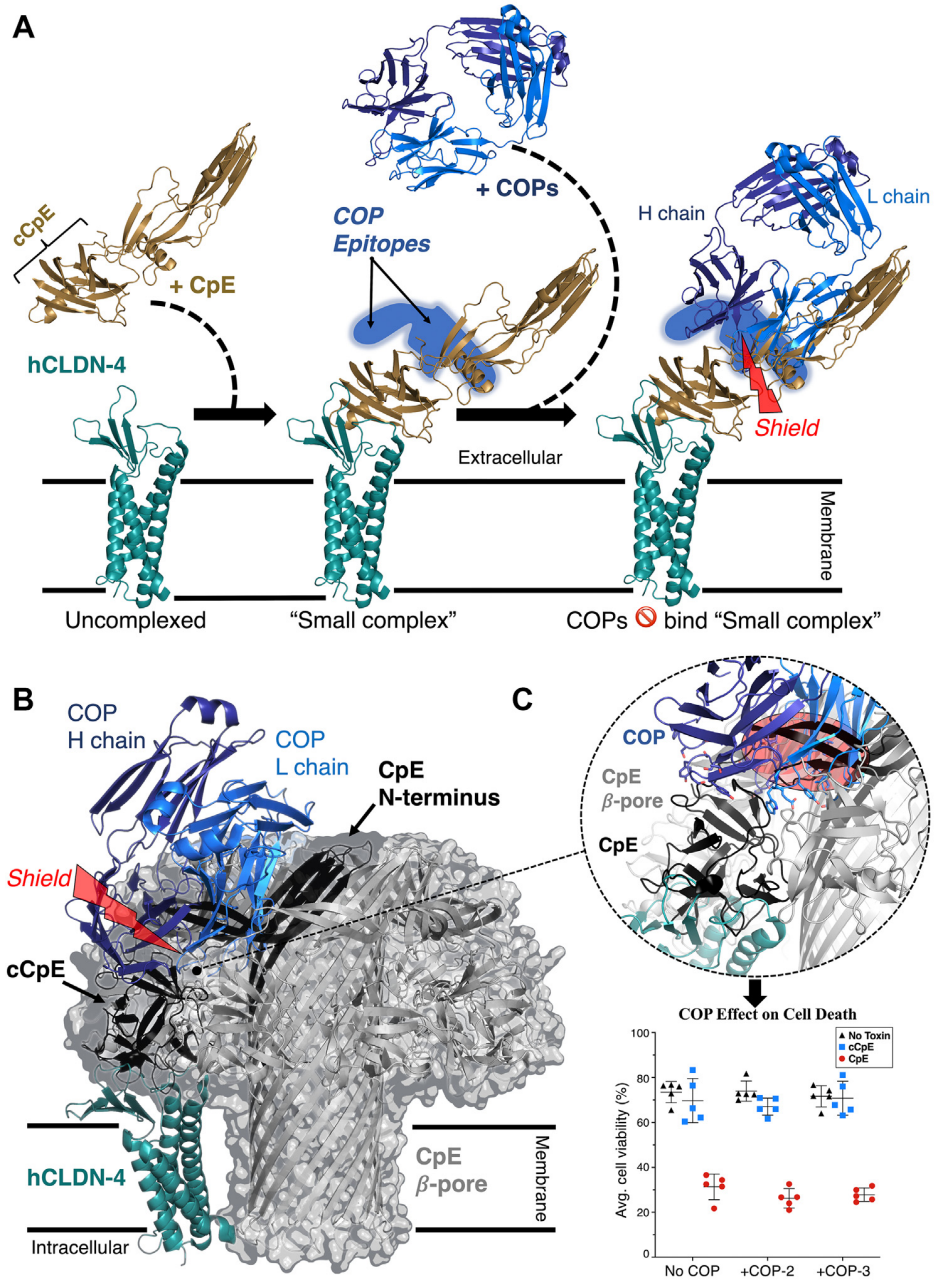
**Models for COP binding to claudin-4/cCpE and the claudin-4–bound CpE *β*-pore.***A*, CpE (*copper*) binding to the ECS of hCLDN-4 (*teal*) forms the “small complex”. The N-terminus of CpE sterically shields the COP (*blue*) binding epitope on cCpE, preventing high-affinity interactions. *B*, model for a CpE *β*-pore (*gray*) based on lysenin with one hCLDN-4 (*teal*) bound to the cCpE domain of a CpE monomer (*black*). Rearrangement of the CpE N-terminus forms the *β*-pore. Despite rearrangement, CpE assembly and its N-terminus sterically shield COP (*blue*) engagement with its binding epitope. *C*, zoom-in on the sterically shielded region shows CDRs from the L and H chains cannot access cCpE. Scatter plot shows the functional effect of COP addition to CpE-induced cytotoxicity of insect cells expressing hCLDN-4. The graph plots the mean and SD based on five readings. Proteins are represented as cartoons or as a translucent surface-encapsulated cartoon in the case of the CpE *β*-pore (*B*). cCpE, CpE’s C-terminal domain; COP, CpE obstructing protein; ECS, extracellular segment.

To predict COP function in the context of CpE-induced cytotoxicity, we created models for the process of COP binding to the claudin-4/CpE “small complex” and for a claudin-4–bound CpE *β*-barrel pore complex, using the cryo-EM structures as guides. We first modeled CpE binding to claudin-4, then COP recognition of the “small complex” (Fig. 6*A*). The model shows in a “small complex” that CpE’s N-terminal domain would sterically shield COP access to their binding epitopes on cCpE. This finding explains our results from biophysical measurements with CpE (Table 1). We next modeled the claudin-4–bound CpE *β*-pore complex (Fig. 6*B*). As there is no structure for the CpE *β*-pore, we used the cryo-EM structure of lysenin, a *β*-pore toxin with homology to CpE to model it (36). Lysenin was chosen because attempts to model the complex using the aerolysin *β*-pore, another homologous protein, did not place claudin perpendicular to the membrane plane. This model reveals that when oligomeric CpE assembles into a *β*-pore complex that COP access to cCpE-binding epitopes would be sterically obstructed even if CpE N-terminal domain rearrangements occur (Fig. 6, *B* and *C*). These structural models explain our *in vitro*–binding data and provide a prediction for the effect of COPs on CpE-induced cytotoxicity *in vivo*.

### Effects of COPs on CpE cytotoxicity

Finally, we used a cell-based assay to validate our model of the claudin-4–bound CpE *β*-pore and to test if COPs affect cytotoxicity. Using *Spodoptera frugiperda* cells that lack endogenous claudins but form tight junction-like strands when expressing claudins on their surfaces, we added COPs, followed immediately by cCpE or CpE, using previously described methods (16, 24, 37, 38). Control wells had no COPs or enterotoxins added. We then measured cell viability by quantifying the amount of cell death instigated by CpE to determine whether COPs altered cytotoxicity (Fig. 6*C*). For cells expressing claudin-4 alone not treated with COPs, we found that cell viability averaged 73.5%. For these cells, COP addition decreased average viability by 0.7%, indicating no COP-induced cytotoxicity. For cells expressing claudin-4 treated with cCpE, which lacks the cytotoxic domain, we found viability averaged 69.7%. Again, for cCpE-treated cells, COP addition decreased average viability only 0.8%. Finally, for cells expressing claudin-4 treated with CpE, we found that cell viability averaged 31.3%, a decrease of >40% compared to untreated and cCpE-treated cells, indicating CpE-induced cytotoxicity. Addition of COPs to CpE-treated cells did not significantly change CpE-induced cell death, decreasing average viability by 4.3%. These results validate our *in silico* model of the claudin-4–bound CpE *β*-pore and establish the effect of COPs to CpE-induced cytotoxicity.

## Discussion

Here, we demonstrate the development of sFabs called COPs, COP-2 and COP-3. We show that COPs bind well to cCpE but not to claudin-4 or CpE; COPs bind cCpE better when bound to claudin-4 than when alone in solution; and that both COPs bind the same epitope of cCpE opposite to where claudin-4 binds (Fig. 1). We also show that COP binding to claudin-4–cCpE complexes yield similar affinities but different kinetics, with COP-2 associating and dissociating more slowly than COP-3 (Table 1). As COPs target and bind cCpE on the opposing surface to its claudin-binding motif, they are therefore capable of binding to any claudin–cCpE complex, making them general yet strategic tools for enabling structures of claudin–cCpE complexes.

As proof of this concept, we determine cryo-EM structures for COP-2 and COP-3 bound to claudin-4–cCpE complexes, which reveal the cCpE-binding epitope and the potential interaction interfaces for the L and H chains of COPs (Fig. 2 and Fig. 3). Our structures and computational analyses show that COP-2 has an 11% larger cCpE/COP interface area, that COP-2 uses chain L CDR-L1 uniquely, and that its binding conforms to or molds cCpEs surface to a greater extent than COP-3, which explains its higher affinity and slower kinetic rates than COP-3. These analyses also reveal a common binding property to COPs where chain H interacts with both sides of a *β*5-*β*6 epitope in a mechanism similar to a caliper brake on a bicycle (Fig. 4, *D* and *H*). Holistically, the cryo-EM structures resemble and thus provide independent validation of the claudin-4–cCpE complex crystal structure.

To validate these moderate resolution cryo-EM structures, we mutate the observed COP-binding epitopes on cCpE and quantify mutant effects to COP binding. For this, we mutated side chains in sequential three to five residue zones. We show that all mutations affect cCpE/COP binding affinity, kinetics, or both (Table 2). Some, like mutants cCpE^3^ and cCpE^4^, display COP-specific differences in their binding and thus pinpoint cCpE residues that are uniquely recognized by COP-2 or COP-3. The changes to COP binding by cCpE mutants *in vitro* validate our cryo-EM structures by attesting to the accuracy of their modeled cCpE/COP interfaces. We further show that models of mutant cCpE–COP complexes guided by our structures elucidate the structural bases of mutant effects to COP binding (SI Fig. S7). In sum, these results provide the likely amino acid determinants and biophysical interaction mechanisms that direct COP-specific recognition of and binding to cCpE.

We further present models for the claudin-4/CpE “small complex” and claudin-4–bound CpE *β*-pore complex in order to understand our structures in a functional context and to predict COP efficacy for use as CPOs (Fig. 6). One model shows that when CpE is bound to claudin-4 in a “small complex”, the N-terminus of CpE sterically shields COP-binding epitopes (Fig. 6*A*). This model is verified by our finding that COP-2 and COP-3 bound CpE with ∼100-fold lower affinity than cCpE (Table 1). Using the homologous *β*-pore–forming toxin lysenin as a benchmark, we also show how CpE may oligomerize to form its membrane-spanning *β*-pore upon binding to claudin-4 (Fig. 6*B*). This model shows that COPs are sterically occluded from accessing their epitopes on cCpE due to CpE oligomeric assembly and N-terminal *β*-pore engagement (Fig. 6*C*). Based on this model, we hypothesized that COPs would be ineffective at preventing CpE-induced cytotoxicity. We test this hypothesis using a cell-based cytotoxicity assay and show that COPs do not prevent nor alter cytotoxicity induced by CpE (Fig. 6*C*). This finding verifies our hypothesis and provides evidence that our model of the CpE *β*-pore is potentially useful to predict the functional effects of structural changes to CpE upon binding claudin-4 and forming a cytotoxic *β*-pore.

**Figure 7:**
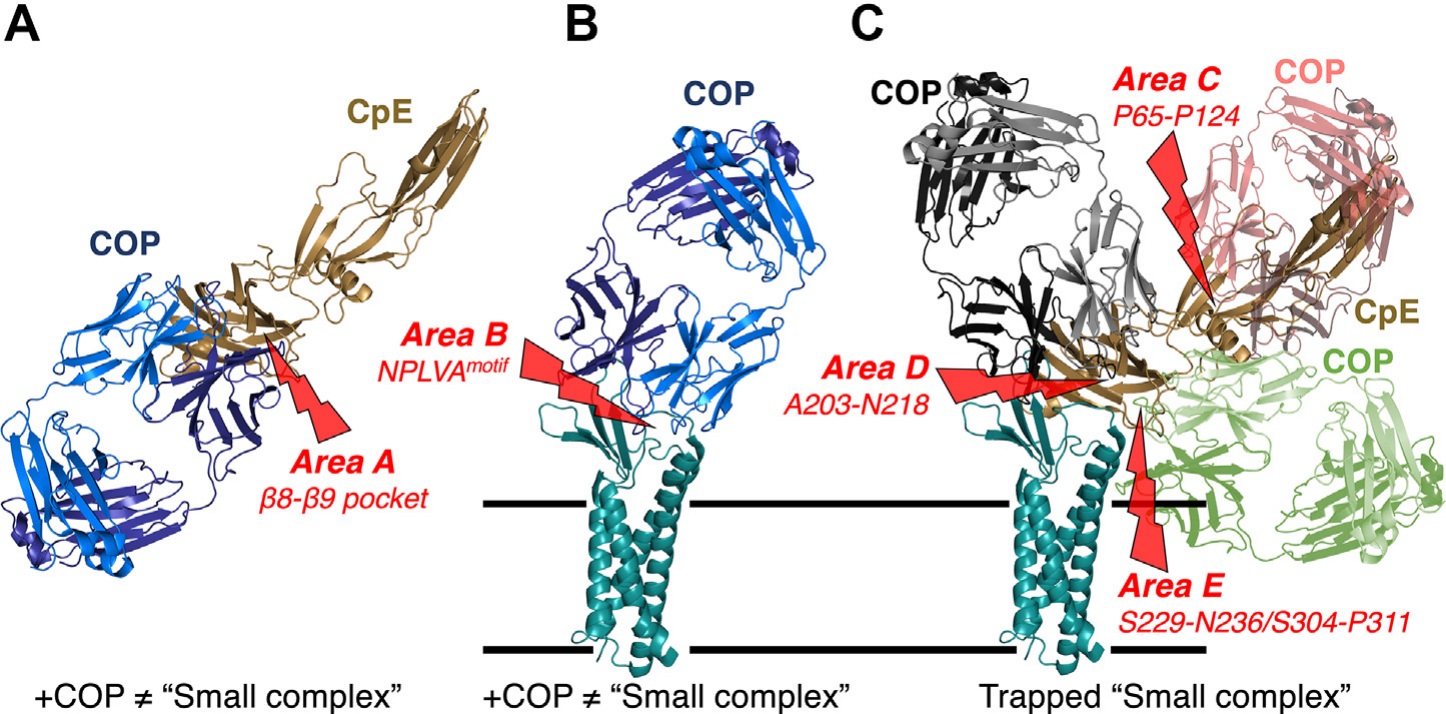
**COP targeting strategies to obstruct CpE cytotoxicity.***A*, COPs (*blue*) that target the cCpE domain of CpE (*copper*) by binding to *Area A*, a solvent-accessible pocket between *β*8 and *β*9 of cCpE that is known to interact with claudin-4’s NPLVA^153^ motif, which is critical for “small complex” formation. *B*, COPs (*blue*) that target hCLDN-4 (*teal*) by binding to *Area B*, the two ECS of claudins, which coordinate to interact with cCpE and form a “small complex”. *C*, COPs (*red*, *black*, *green*) that target CpE (*copper*) by binding to *Areas C*, *D*, and *E*. *Area C* comprises Pro65-Pro124 of CpE’s N-terminus, which contains a purported Thr92-Gly105 ⍺-helix used for *β*-pore formation. *Area D* comprises Ala203-Asn218 of cCpE, which contains its *β*1 strand and ⍺1 helix. *Area E* comprises Ser229-Asn236 and Ser304-Pro311 of cCpE, which contain two loops that connect strands *β*2 to *β*3 and *β*8 to *β*9. These three areas may be used for CpE assembly and thus COP binding could obstruct this assembly, trapping “small complexes” that are inert and not cytotoxic. cCpE, CpE’s C-terminal domain; COP, CpE obstructing protein; ECS, extracellular segment.

Based on these data and results, we propose a framework for using COP-like molecules to target and obstruct CpE cytotoxicity (Fig. 7). We envision three strategies based on the proposed sequence of events that lead to CpE *β*-pore formation (17, 18). These include developing COPs that 1) obstruct formation of claudin-4/CpE “small complexes”, 2) stabilize the native fold of CpE’s N-terminus to prevent its structural rearrangement that induces cytotoxicity via *β*-pore formation, and 3) obstruct CpE oligomerization prior to *β*-pore assembly. Using our models for the “small” and claudin-4–bound CpE *β*-pore complexes, we identify five areas where binding by COP-like sFabs could obstruct CpE binding, conformational changes, or oligomerization, potentially preventing CpE-based cytotoxicity. Area A is a surface pocket between *β*8 and *β*9 on the cCpE domain of CpE (Fig. 7*A*); and area B comprises the two ECS of claudins and includes the NPLVA^153^ motif that imparts high-affinity CpE binding (Fig. 7*B*) (16). Development of COPs that bind area A or B would inhibit formation of the “small complex” by obstructing areas known to facilitate claudin-4–cCpE interactions, thus preventing CpE prepore and *β*-pore complex formation (Strategy 1). Area C is a region of CpE’s N-terminus that may structurally rearrange upon CpE assembly forming a long, antiparallel *β*-barrel to create the membrane-penetrating *β*-pore (Fig. 6*B* and Fig. 7*C*). Discovery of COPs that bind area C may stabilize CpE’s N-terminus to disable its *β*-pore–forming conformational transition (Strategy 2)—they may also obstruct CpE oligomerization (Strategy 3). Finally, area D includes strand *β*1 and helix ⍺1 of cCpE; while area E comprises two loops that connect *β*2 to *β*3 and *β*8 to *β*9 (Fig. 7*C*). Based on our models, areas D and E represent additional areas where COP binding could sterically obstruct CpE oligomerization. In summary, our models reveal that 1) for COP binding to areas A or B to be effective at obstructing cytotoxicity, they will need to be present before or during CpE production to prevent “small complex” formation and 2) COP binding to areas C, D, or E would not prevent “small complex” formation and thus could be effective postproduction of CpE but would need to be present prior to CpE’s oligomeric assembly of its *β*-pore. This proposed structure-based framework can inform therapeutic strategies that utilize yet to be developed COPs to target CpE, claudin-4, or claudin-4–CpE complexes. COPs that bind the above-mentioned areas could obstruct CpE *β*-pore formation and thus prevent CpE-linked cytotoxicity. Such molecules could be potent therapeutics, capable of treating CpE-linked gastrointestinal illnesses, afflict millions of humans and domesticated animals globally, resulting in large economic burdens and losses in quality of life.

## Conclusions

In this study, we progress a novel workflow for structural determination of claudin–enterotoxin complexes using 200 kV cryo-EM instrumentation that yields 4 to 7 Å resolutions. Our approach is amenable to other cCpE-binding claudins in a variety of membrane mimetics and is enabled by COPs, which add mass, rigidity, and act as fiducial marks (SI Fig. S8). COPs are versatile, capable of being used for cryo-EM and/or as crystallization chaperones for X-ray diffraction. Although the cryo-EM workflow described here produces modest resolution structures, it must be considered that these complexes are small by cryo-EM standards at 35 kDa. We estimate that their already detailed maps could be improved to 3 to 4 Å using 300 kV microscopes (SI Fig. S4). Achieving these resolutions would equal current results from crystallography of claudin–cCpE complexes and have the added advantage of enabling structure determination of complexes recalcitrant to crystallization or complexes that crystallize but diffract X-rays weakly. Thus, COPs are multiuse tools to expand structural knowledge of other claudins that bind enterotoxins. For this and their potential obstructing capacities, COPs and COP-like sFabs may provide new insights useful for developing treatments for CpE-based diseases or to aid design of novel cCpE- and CpE-based therapeutics that modulate tight junction barriers.

## Experimental procedures

### Claudin-4 and enterotoxin expression and purification

Methods followed those described previously (1,6, 24). Briefly, claudin-4, cCpE, and CpE with C-terminal decahistidine tags preceded by thrombin cleavage sites (claudin-4-His_10_, cCpE-His_10_, and CpE-His_10_) were cloned into pFastBac1 (ThermoFisher) and expressed in Tn5 (*Trichoplusia ni*, High Five, Expression Systems, LLC). Cell pellets resuspended in lysis buffer (50 mM Tris pH 8.0, 150 mM NaCl, 1 mM PMSF, and EDTA-free SigmaFast protease tablets (Sigma)) were sonicated, supplemented with 1 M NaCl, then ultracentrifugation at 100,000×*g* for 1 h. For enterotoxins, the supernatant was saved, 15 mM imidazole was added along with NiNTA resin, and the solution was incubated for 12 h at 4 °C. For claudin-4, the supernatant was removed, and the membrane pellet was resuspended in lysis buffer and the protein was solubilized with 1% (w/v) DDM (Anatrace) and 0.04% cholesteryl hemisuccinate (Anatrace) overnight at 4 °C. Insoluble protein was removed by ultracentrifugation at 100,000×*g* for 30 min, and the supernatant was treated with 15 mM imidazole along with NiNTA resin, and the solution was incubated for 12 h at 4 °C. For claudin-4, the bound protein was captured and washed with five column volumes of buffer A (50 mM Tris pH 7.4, 500 mM NaCl, 25 mM imidazole, and 0.087% DDM) and buffer B (buffer A containing 300 mM NaCl and 40 mM imidazole). Buffer T (50 mM Tris pH 8.0, 150 mM NaCl and 0.04% DDM) was used to release and capture proteins from the resin after treatment with thrombin. For enterotoxins, purification was similar to claudin-4 except DDM was not added to buffers. These proteins were then used for sFab panning or biochemical, biophysical, and structural analyses. For enterotoxins used for binding studies, the proteins were eluted off of NiNTA using elution buffer (buffer T containing 400 mM imidazole) to keep the His_10_ tag. Eluted enterotoxin-His_10_ was dialyzed in SEC buffer (10 mM Hepes pH 7.4, 100 mM NaCl, and 4% glycerol), concentrated to 1 mg/ml, then flash-frozen in liquid nitrogen and stored at −80 °C until use.

### Generation and validation of COPs using phage display

DDM-solubilized claudin-4 was biotinylated using N-hydroxysuccinimide polyethylene glycol biotin (NHS-PEG4-biotin, ThermoFisher) by mixing 5.6 μM claudin-4 with 16.8 μM NHS-PEG4-biotin, followed by incubation on ice for 2 h. Excess cCpE with the His_10_ removed was added at a ratio of 1:1.5 (moles:moles), then free biotin and unbound cCpE was removed by loading the sample onto a Superdex 200 Increase 10/300 Gl (Cytiva) equilibrated in SEC buffer containing 0.04% DDM. The sFab panning was performed using the sFab library E (32, 33). Binding was assayed in selection buffer (25 mM Hepes pH 7.4, 150 mM NaCl, and 1% bovine serum albumin). A first round of panning was performed manually using 200 nM of biotinylated claudin-4/cCpE in DDM immobilized onto magnetic beads, and following three washes with Selection buffer, the beads enriched for phage-expressing claudin-4/cCpE-specific sFabs were used to infect log-phase *Escherichia coli* XL1-Blue cells. Phages were amplified overnight in 2xYT media supplemented with 100 μg/ml ampicillin and M13-KO7 helper phage (10^9^ pfu/ml). Selection stringency was then increased by four additional rounds of panning using decreasing claudin-4/cCpE concentrations down to ∼20 nM. For each round, the amplified phage pool from each preceding round was used as the input. For rounds two to five, panning was performed semiautomatically using a Kingfisher magnetic beads handler (ThermoFisher). Nonspecific binding of sFabs to detergent was reduced by using >0.87% DDM in later rounds. Bound phage particles were removed by elution from beads using 1% Fos-choline-12.

The initial validation was performed by single-point phage ELISA using individual clones from later selection rounds. ELISAs were performed in 96-well plates (Nunc) coated with 2 μg/ml neutravidin and blocked with selection buffer. *E. coli* XL1-Blue colonies–containing phagemids were used to inoculate 400 μl 2xYT media containing 100 μg/ml ampicillin and 10^9^ pfu/ml M13-KO7 helper phage. Phages were amplified overnight in 96-well deep-well blocks at 37 °C with shaking at 280 rpm. Phages were then diluted 1:10 into selection buffer and assayed against claudin-4/cCpE in DDM or buffer containing only DDM micelles. Biotinylated claudin-4/cCpE was immobilized at room temperature for 30 min, then incubated with phage dilutions. Bound phage were detected with TMB substrate (ThermoFisher) following a 30 min incubation with horseradish peroxidase–conjugated anti-M13 monoclonal antibody (GE Healthcare). Absorbance was measured at 450 nm after quenching the reaction with 1.0 M HCl. Wells containing 1% DDM were used to detect nonspecific binding.

### COP expression and purification

Two sFabs termed COP-2 and -3, from phage ELISA, were selected and sequenced at the University of Chicago Comprehensive Cancer Center DNA Sequencing facility. Unique clones for each COP were subcloned in pRH2.2 using the In-Fusion Cloning kit (Takara Bio). Sequence-verified COPs were transformed into *E. coli* BL21-Gold cells (Agilent) and then used to inoculate overnight cultures. The inoculates were then used to seed 1 l of 2xYT media containing 100 μg/ml ampicillin. Cultures were grown to an A_600_ of 0.8, induced for 4 h at 37 °C, then cells were harvested using centrifugation. Cell pellets were resuspended in COP lysis buffer (20 mM Hepes pH 7.4, 150 mM NaCl, 0.5 mM MgCl_2_, 1 mM PMSF, and 1 μg/ml DNase I). Cells were sonicated and lysates were incubated at 60 °C for 30 min to remove proteolyzed fragments. Samples were cooled rapidly on ice then cleared by centrifugation. Supernatants were filtered by 0.45 μm and loaded onto a 5 ml HiTrap MabSelect SuRe column (GE Healthcare) equilibrated with COP Wash buffer (20 mM Hepes pH 7.4 and 500 mM NaCl). The column was washed with 10 column volumes of COP Wash buffer, and COPs were eluted with 0.1 M acetic acid. Eluted COPs were loaded onto a 1 ml Resource S column (GE Healthcare) equilibrated with COP buffer A (50 mM sodium acetate pH 5.0) and washed with 10 column volumes of this buffer. The COPs were eluted by linear 0 to 50% gradient with COP buffer B (COP buffer A containing 2 M NaCl). COP-containing fractions were pooled and dialyzed overnight at 4 °C in SEC buffer.

### Biochemical characterization of COP binding

We used post-NiNTA–purified untagged claudin-4 and enterotoxins and postaffinity-purified COPs for these analyses. For claudin-4/COP and enterotoxin/COP studies, 50 μg enterotoxins was used and excess COPs were added at a 1:1.2 M ratio, incubated at room temperature for 1 h, concentrated, 0.2 μm filtered, then loaded onto a Superdex 200 column equilibrated in SEC buffer containing 0.04% DDM. For claudin-4/enterotoxin/COP studies, 50 μg claudin-4 was used and excess enterotoxins were added at a 1:1.2 M ratio, then COPs were added at a 1:1 M ratio to enterotoxins. Complexes were incubated, concentrated and filtered, and loaded onto a Superdex200 column equilibrated in SEC buffer and 0.04% DDM. Complex formation was assessed by observed decreases in the elution times of the uncomplexed peak fractions. Peak fractions containing complexes were pooled, unboiled, and evaluated for the presence of each protein by SDS-PAGE using 4 to 20% agarose gradient gels.

### Biophysical characterization of COP binding using BLI

BLI analyses were performed at 25 °C at an acquisition rate of 5 Hz averaged by 20 using an Octet© BLItz System (FortéBio/Sartorius), with assays designed and setup using Blitz Pro 1.3 Software. A typical experiment consisted of the following steps: sensor equilibration (30 s), protein loading (200 s), baseline (60 s), and association and dissociation (300 s each). For the loading step, 4 μl of proteins were loaded in the drop holder, while all other steps were performed in transparent 600 μl microtubes using 250 μl sample volumes. All measurements were performed in SEC buffer containing 0.04% DDM. For cCpE/COP studies, 5 μM (70 μg/ml) of WT cCpE-His_10_ was loaded on NiNTA (Dip and Read) sensors then dipped into a 0 to 1 μM range of four concentrations of COPs for the association steps. For claudin-4/enterotoxin/COP studies, 5 μM DDM-solubilized claudin-4 biotinylated with NHS-PEG4-biotin as before was precomplexed with excess WT or mutant cCpE or CpE without a His_10_ at a ratio of 1:2 (moles:moles) for 1 h at room temperature. Claudin-4–enterotoxin complexes were loaded on Streptavidin-SA (Dip and Read) sensors and the measurements were performed as above using 0 to 30 μM range of concentrations depending on the analyte. For claudin-4/mutant cCpE studies, 5 μM of biotinylated claudin-4 in DDM was immobilized on SA sensors and measurements were performed as above using a 0 to 0.25 μM concentration range of cCpEs. For all studies, measurements were repeated in at least duplicate, and the time courses for association and dissociation were fit to one-site–binding model using the BLItz Pro 1.3 Software. No major nonspecific binding of COPs to unloaded NiNTA or streptavidin sensors were detected at concentrations up to 30 μM.

### Cryo-EM sample preparation

DDM-solubilized claudin-4 was exchanged into 2,2-didecylpropane-1,3-bis-*β*-D-maltopyranoside (LMNG, Anatrace) or amphipol A8-35 (Anatrace). For exchange into LMNG, the protein bound to NiNTA resin was sequentially washed with buffer A, B, and T containing 0.087, 0.087, and 0.04% LMNG, respectively, before releasing the bound protein from resin via thrombin cleavage. To prepare sample in amphipol, 1 mg of postthrombin-digested claudin-4 in DDM was treated with 4 mg of amphipol (1:4 w/w) for 2 h before removing detergent via addition of 400 mg SM-2 biobeads (Bio-Rad). Excess cCpE was added to each claudin-4 sample at a molar ratio of 1:1.5, incubated at room temperature for 1 h, then excess COPs were added at a 1.5:1 ratio to cCpE. After 1 h at room temperature, each sample was concentrated, 0.2 μm filtered, and then loaded onto a Superdex 200 Increase 10/300 Gl equilibrated in SEC buffer without glycerol but with 0.003% LMNG or no detergent for amphipol samples. Peak fractions from SEC-containing claudin-4–cCpE–COP complexes were collected and concentrated to 6 to 8 mg/ml for use in cryo-EM analyses.

Grids for cryo-EM analyses were prepared using a Vitrobot Mark IV (ThermoFisher) plunge freezing apparatus. Aliquots (3 μl) of claudin-4–cCpE–COP complexes in LMNG or amphipol were applied to Quantifoil R1.2/1.3200 mesh grids that were glow-discharged for 45 s at 15 mA in a Pelco easiGlow (Ted Pella Inc) instrument. Protein solutions were applied to grids at 4 °C and 100% relative humidity and allowed to adsorb on the grid for ∼30 s before blotting. Grids were blotted for 5 to 8 s with a blot force of 1 and plunge frozen into liquid ethane cooled by liquid nitrogen. Grids were stored in liquid nitrogen prior to imaging.

### Cryo-EM data collection and processing

Cryo-EM data collection was performed on a Talos Arctica (ThermoFisher) equipped with a Falcon III (ThermoFisher) direct electron detector at Michigan State University. Grids were screened for thin ice and good particle distribution, and data collection was performed using EPU software (ThermoFisher). Movies of claudin-4/cCpE/COP-2 in LMNG were collected on the Falcon III detector in counting mode at 92000× magnification with a pixel size of 1.12 Å, a defocus range of 0.8 to 2.6 μm, and a total dose of ∼32 electron/Å^2^ fractionated over 51 total frames. Movies of claudin-4/cCpE/COP-3 in amphipol were collected at 120000× magnification with a pixel size of 0.87 Å, a defocus range of −1 to −2.2 μm, and a total dose of ∼40 electron/Å^2^ fractionated over 42 total frames.

All micrograph and particle processing was performed in CryoSPARC (39). Patch-motion correction and patch-CTF correction were used to correct for beam-induced motion and calculate CTF parameters from the motion-corrected micrographs. Blob-based template picking followed by 2D classification was used to generate templates that were subsequently used for template-based particle picking. Particles identified from this template-based picking procedure were subjected to several rounds of 2D classification, followed by *ab initio* 3D reconstruction, heterogeneous refinement, and nonuniform refinement. In the case of the COP-3 complex, local refinement using a soft mask around cCpE and COP-3 was also used to improve resolution at the complex’s interface.

### Cryo-EM model building, refinement, and structure determination

Using postprocessing maps, we first built the structure of claudin-4/cCpE/COP-2 using the 6.9 Å map and the crystal structure of claudin-4 in complex with cCpE (PDB ID 7KP4) (16). 7KP4 was manually docked by placing the four TMs of claudin-4 into density present in the LMNG micelle using Coot (40). TM placement was validated using density corresponding to bulky side chains, like Trp18 in TM1 of claudin-4. Initial placement of the TM region showed that only minor alterations to claudin-4’s ECS and cCpE would be required to fit well within the cryo-EM map. For this part of the structure, the map volume allowed placement of most claudin-4 and cCpE residues, with the exception of claudin-4’s C-terminus and cCpE’s N-terminus. We next built COP-2 by first using a high-resolution crystal structure of an sFab (PDB ID 6CBV). COP-2 was manually docked into the additional cryo-EM density using Coot, and then divergent residues of COP-2 were manually mutated from the 6CBV template using sequence alignments as a guide (41). Once the three proteins were fit manually, each of the four protein chains were rigid body refined in Coot to place the structure within the cryo-EM map volume. Model building was done using Coot and once complete, the structure was further refined using a combination of molecular dynamics (MD) flexible fitting simulations using Namdinator followed by real-space refinement of the model into the cryo-EM density map using Phenix phenix.real_space_refine (42, 43). The final structural model required secondary structure and Ramachandran restraints to optimize the model-to-map fit and overall geometry. SI Table S1 shows data collection, refinement, and validation statistics for the claudin-4/cCpE/COP-2 structure.

The structure of claudin-4/cCpE/COP-3 was determined using both a masked and unmasked strategy, which produced *focused* and *whole* maps that resolved to 3.8 and 5.0 Å, respectively. A mask was applied to COP-3 and cCpE of the complex in the former case, while all three proteins were included (unmasked) in the latter. The final model of the claudin-4–cCpE–COP-2 complex was manually docked, then COP-3 was made from COP-2 by mutating divergent residues using sequence alignments. Each protein chain in the complex was then individually refined as rigid bodies into the cryo-EM maps using Coot. While the *focused* map had added features for COP-3 and cCpE, because of the mask, it lacked density for claudin-4 and refinements using the three-protein complex resulted in poor model-to-map fits. Thus, all three proteins were built and fit manually within the *whole* map volume using Coot and the final model was refined using Namdinator and Phenix phenix.real_space_refine using secondary structure and Ramachandran restraints (42, 43). The deposited PDB ID 7TDN is a result of refining against the *whole* cryo-EM map. SI Table S1 shows data collection, refinement, and validation statistics for the claudin-4/cCpE/COP-3 structure.

The programs used to visualize and build the structures included Coot, PyMOL, and Chimera, refined using Phenix, and Figures were made using PyMOL—using the SBGrid Consortium Software Suite (40, 44, 45, 46, 47).

### Mutagenesis of cCpE

The cCpE-His_10_ was altered using site-directed mutagenesis. Mutants were generated with the following forward and equivalent reverse primers:

cCpE^1^ 5′-gcgcggatccgccaccgcatcaacggacattatgaaagaaatcctcgac-3’;

cCpE^2^ 5′-ccatgtcaacggacattgaaaaagaagccgccgccttagctgctgcaacagaacgc-3’;

cCpE^2L^ 5′-ccatgtcaacggacattgaaaaagaagccgccgccgccgctgctgcaacagaacgc-3’;

cCpE^3^ 5′-gttgactttaacatttactccaacgccgccgctgcccttgtcaaactcgaacaatcgctc-3’;

cCpE^4^ 5′-catttactccaacaacttcaataacgctgccaaagccgcacaatcgctcggagatggtg-3’. Expression and purification of all cCpE-His_10_ mutants were identical to those of WT cCpE-His_10_.

Amino acid sequence alignments comparing mutant cCpE to WT are provided to aid interpretation.

**Table:** 

cCpE^1^	CCpE^WT^ cCpE^Δ192-196^	^192^STDIEKEILDLAAATERLN^210^ ^197^KEILDLAAATERLN^210^
cCpE^2^	cCpE^WT^ cCpE^Ala199-201^	^192^STDIEKEILDLAAATERLN^210^ ^192^STDIEKE*AAA*LAAATERLN^210^
cCpE^2L^	CCpE^WT^ cCpE^Ala199-202^	^192^STDIEKEILDLAAATERLN^210^ ^192^STDIEKE*AAAA*AAATERLN^210^
cCpE^3^	cCpE^WT^ cCpE^Ala267-270^	^260^DFNIYSNNFNNLVKLEQ^276^ ^260^DFNIYSN*AAAA*LVKLEQ^276^
cCpE^4^	cCpE^WT^ cCpE^Ala271-272/274-275^	^260^DFNIYSNNFNNLVKLEQ^276^ ^260^DFNIYSNNFNN*AA*K*AA*Q^276^

### Structural modeling of claudin-bound CpE pore

A model of claudin-4/CpE was made by superposing CpE (PDB ID 3 AM2) onto cCpE from our cryo-EM structure of the COP-2 complex, making a claudin-4/CpE/COP model. To approximate the CpE *β*-pore, we modeled it after lysenin, a *β*-pore toxin with homology to CpE, using an available cryo-EM structure (PDB ID 5GAQ) (36, 48). Using a crystal structure of the lysenin monomer (PDB ID 3ZXD), we superposed 3ZXD onto one monomer of the 5GAQ nonamer and then superposed the CpE portion of our claudin-4/CpE/COP model onto 3ZXD (49). We next removed 3ZXD. This process integrated claudin-4/CpE/COP into lysenin, making the model for the claudin-4–bound CpE *β*-pore. These models were used to predict COP influence on CpE-induced cytotoxicity.

### Cell-based cytotoxicity assay

Recombinant baculoviruses–containing claudin-4-His_10_ were produced using established methods and the cytotoxicity assay was performed using methods previously described (16). To 18 wells of a 24-well cell culture plate, 0.5 ml of 1.0 x 10^6^ adherent Sf9 (*S. frugiperda*, Expression Systems) cells were added. After 1 h at 27 °C, virus–containing claudin-4-His_10_ was added at a MOI of 1.0. Cultures were rocked gently and then placed at 27 °C for 48 h. The 18 wells were divided into three, 6-well groups. To duplicate wells of group one and three, 22 μg (0.9 μM) of COP-2 was added; to a second pair of wells, 22 μg (0.9 μM) of COP-3 was added; and to the third pair of wells, no COP was added. To duplicate wells of group two, 100 μg (4 μM) of COP-2 was added; to a second pair of wells, 100 μg (4 μM) of COP-3 was added; and to the third pair of wells, no COP was added. For group one, nothing more was added; this group contained only cells expressing claudin-4-His_10_. For group two, 25 μg (3.3 μM)–purified cCpE in SEC buffer was added to the culture medium. For group three, 12.5 μg (0.7 μM) CpE in SEC buffer was added to the culture medium. The COP amounts added correspond to 1.2-fold molar excess of COP to enterotoxin. After addition of COPs and/or enterotoxins, Sf9 cells were placed at 27 °C for 12 h, then CpE cytotoxicity was quantified using a cell viability analysis. This was done by gently removing 250 μl of Sf9 cells from each well, centrifuging at 200×*g* for 2 min, removing 200 μl of media, then adding 50 μl of 0.04% trypan blue. After 5 min, 10 μl of stained cells were transferred to a Countess cell counting chamber slides and counted automatically using a Countess 3 automated cell counter (ThermoFisher). Each well was counted at least two times with one well counted three times (representing five readings per sample, 45 for nine samples). Viability counts consisted of dividing the total number of live cells (unstained) by the total number of cells. Average cell viability for enterotoxin-treated cells was compared to Sf9 cells expressing claudin-4 that were not treated with either enterotoxins or COPs. Data was plotted as a mean with SD in GraphPad Prism 9 for macOS.

## Data availability

The cryo-EM structure of claudin-4/cCpE/COP-2 has accession code 7TDM in the Protein Data Bank (PDB), and cryo-EM maps of this complex have been deposited to the Electron Microscopy Data Bank under accession code EMD-25834. The cryo-EM structure of claudin-4/cCpE/COP-3 has PDB accession code 7TDN, and cryo-EM maps of this complex have been deposited to the Electron Microscopy Data Bank under accession code EMD-25835 (whole) and EMD-25836 (focused).

## Supporting information

This article contains Supporting information (50).

## Conflict of interest

The authors declare that they have no conflicts of interest with the contents of this article.
