# Supplementary material for: Development, structure, and mechanism of synthetic antibodies that target claudin and Clostridium perfringens enterotoxin complexes
Source: J Biol Chem. 2022 Aug 9;298(9):102357. doi: 10.1016/j.jbc.2022.102357 (PMC9463536; doi:10.1016/j.jbc.2022.102357)
Supplement: Supporting Information [file mmc1.docx]

**SUPPORTING INFORMATION (SI)**

**Development, structure, and mechanism of synthetic antibodies that target claudin and *Clostridium perfringens* enterotoxin complexes**

Benjamin J. Orlando^1^, Pawel K. Dominik^2,a^, Sourav Roy^3^, Chinemerem P. Ogbu^3^, Satchal K. Erramilli^2^, Anthony A. Kossiakoff^2^, and Alex J. Vecchio^#,3,*^

^1^Department of Biochemistry and Molecular Biology, Michigan State University, East Lansing, MI, 48824 USA

^2^Department of Biochemistry and Molecular Biology, University of Chicago, Chicago, IL, 60637 USA

^3^Department of Biochemistry, University of Nebraska-Lincoln, Lincoln, NE, 68588 USA

**8 Supporting Figures**

**1 Supporting Table**

**
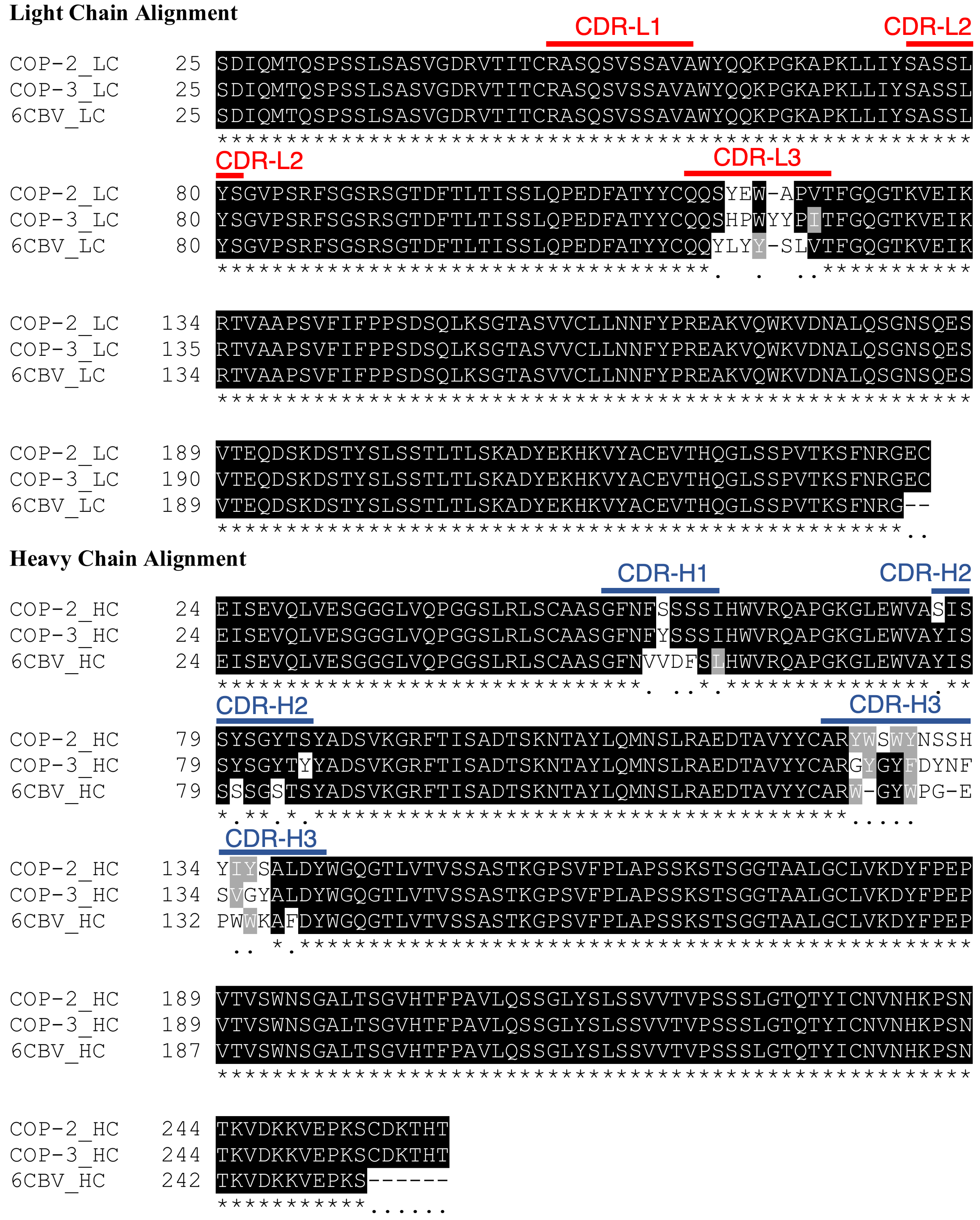
Figure S1. Sequence alignment of COPs.** The L and H chains of COP-2 and COP-3 were sequenced and aligned against the sequence of a generic sFab, PDB ID 6CBV, using T-Coffee ([51](#_ENREF_51)). Highlighted in the sequences are the three CDRs from the L chain (red) and three CDRs from the H chain (blue).

**
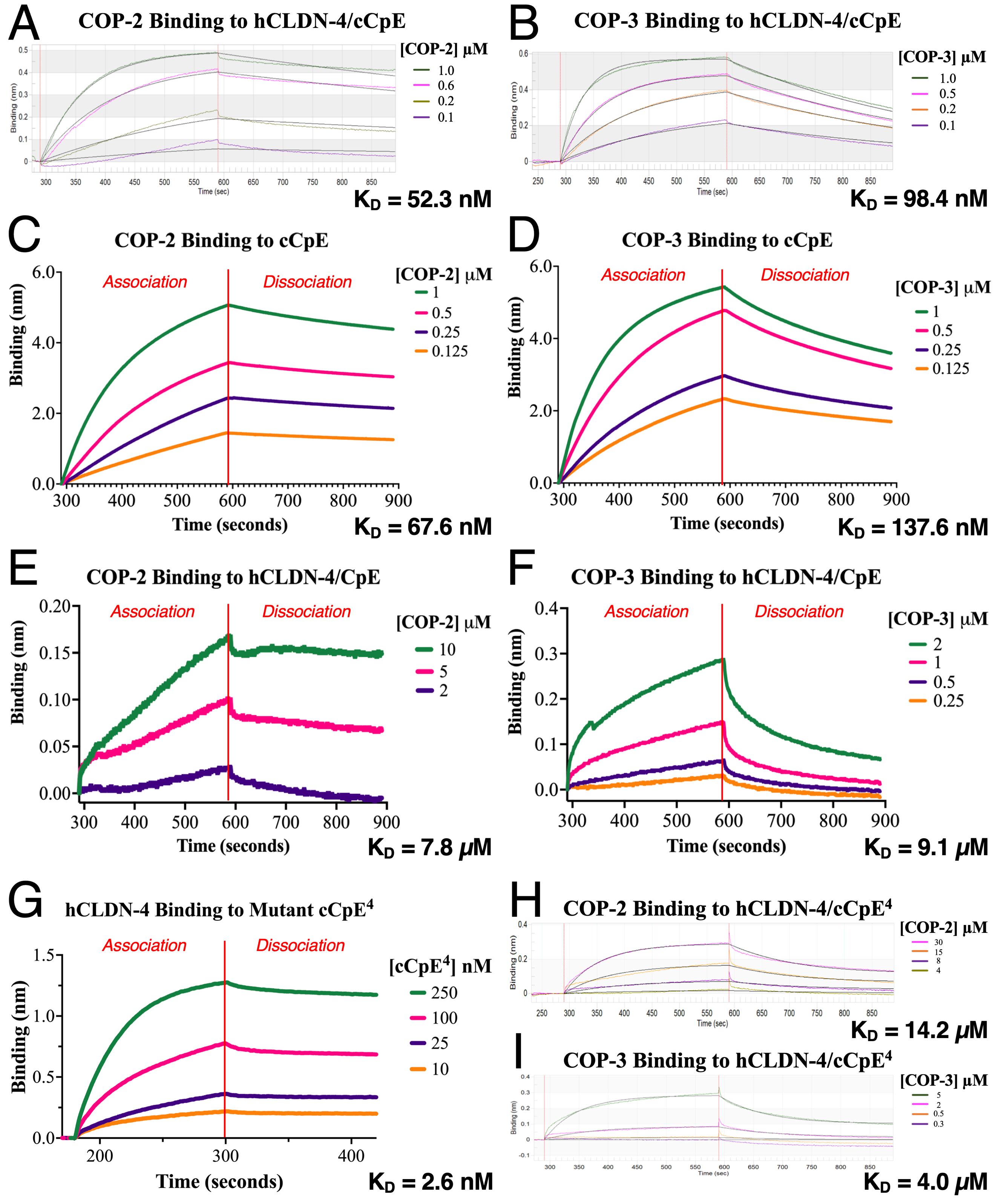
Figure S2. Biophysical binding measurements using BLI.** (A) COP-2 and (B) COP-3 binding to biotinylated human claudin-4 (hCLDN-4)/cCpE complexes immobilized on streptavidin (SA) biosensors. Data (various colors) are overlaid with best fit lines (black). This data is the same as from Fig. 2B and 3B. (C) COP-2 and (D) COP-3 binding to cCpE-His_10_ immobilized on NiNTA biosensors. (E) COP-2 and (F) COP-3 binding to biotinylated hCLDN-4/CpE complexes immobilized on SA biosensors. (G) Mutant cCpE^4^ binding to biotinylated hCLDN-4 using SA biosensors. Analyte concentrations are shown from high to low (various colors) and a red solid line depicts the transition from association to dissociation phase. (H) COP-2 and (I) COP-3 binding to biotinylated hCLDN-4/cCpE^4^ complexes on SA biosensors. A-B and H-I is raw data from BLItz Pro 1.3 Software. For C-G, sensorgrams were re-plotted in Prism 9. All sensorgrams represent a single experiment set from duplicate measurements.

**
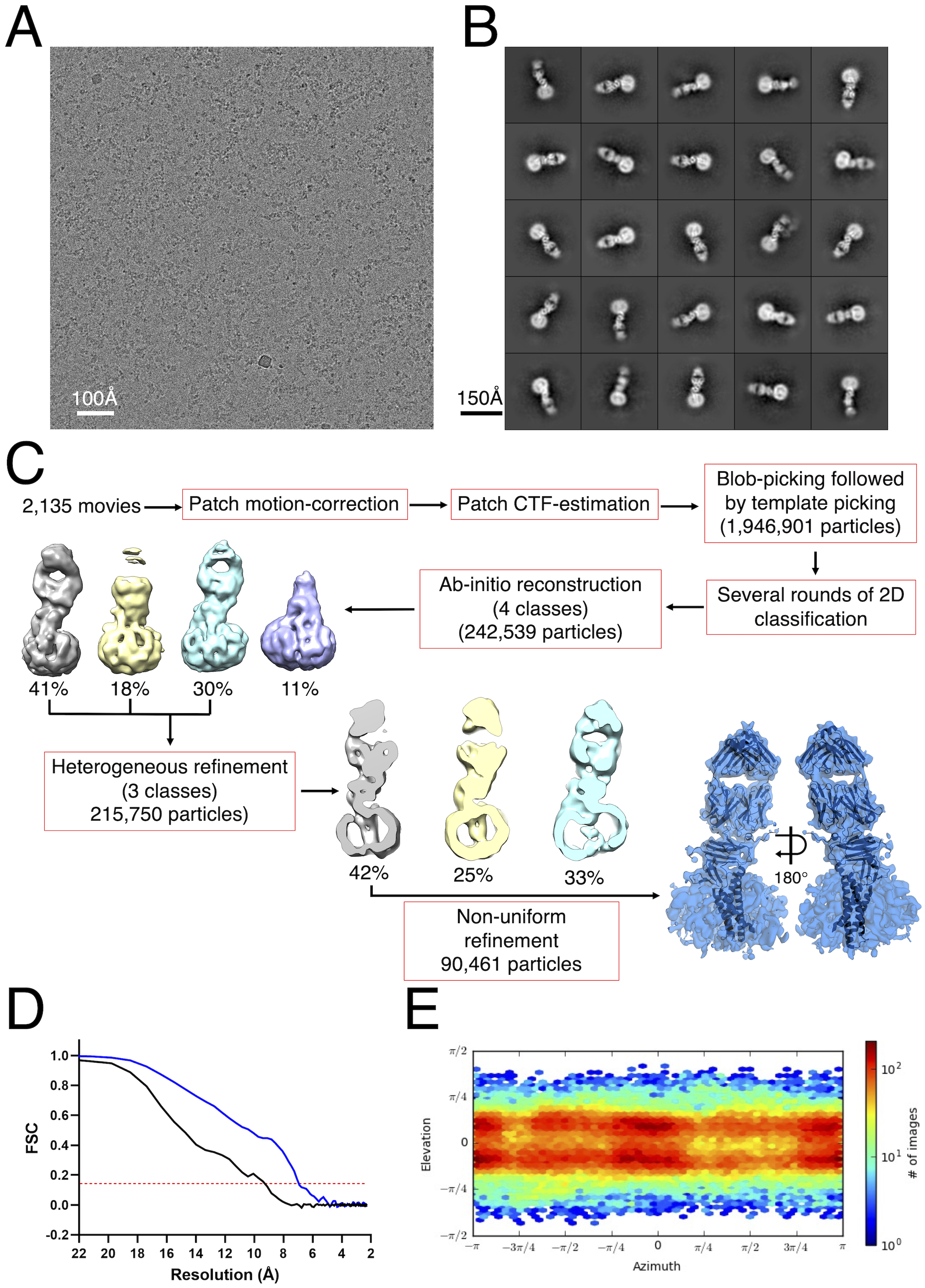
**

**Figure S3. Cryo-EM data processing for claudin-4/cCpE/COP-2 complex.** (A) Electron micrograph showing the distribution of claudin-4/cCpE/COP-2 complexes in ice. (B) Representative 2D class averages of claudin-4/cCpE/COP-2 complexes showing the clear binding of the COP-2 sFab, and signal for TMs within the LMNG detergent micelle. (C) Cryo-EM data processing workflow. (D) Fourier Shell Correlation (FSC) curves from gold-standard refinement. The 0.143 FSC cutoff is indicated by a dashed red line. The black line indicates FSC without a mask applied, and the blue line indicates FSC with a tight mask applied around the protein and detergent micelle. (E) Angular distribution of particles in the final refinement, demonstrating an absence of pure top views.

**Table S1 Cryo-EM data collection, refinement and validation statistics**

|  | **claudin-4/cCpE/**  **COP-2**  EMDB-25834  PDB ID 7DTM | **claudin-4/cCpE/**  **COP-3**  EMDB-25835 (*whole*)  PDB ID 7DTN | **claudin-4/cCpE/**  **COP-3**  EMDB-25836 (*focused*) |
| --- | --- | --- | --- |
| **Data collection and processing** |  |  |  |
| Magnification | 92,000 | 120,000 | 120,000 |
| Voltage (keV) | 200 | 200 | 200 |
| Electron exposure (e–/Å^2^) | 32.0 | 39.6 | 39.6 |
| Defocus range (μm) | 0.8 - 2.6 | 1.0 - 2.2 | 1.0 - 2.2 |
| Pixel size (Å) | 1.12 | 0.871 | 0.871 |
| Symmetry imposed | C1 | C1 | C1 |
| Number of micrographs | 2,135 | 5,132 | 5,132 |
| Initial particle images (no.) | 1,946,901 | 3,758,631 | 3,758,631 |
| Final particle images (no.) | 90,461 | 305,927 | 305,927 |
| Map resolution (Å)  FSC threshold | 6.9  0.143 | 5.0  0.143 | 3.8  0.143 |
|  |  |  |  |
| **Refinement** |  |  |  |
| Initial model used (PDB ID) | 7KP4 | 7DTM | *N/A* |
| Model resolution (Å)  FSC threshold | 6.1  0.143 | 4.5  0.143 | *N/A*  *N/A* |
| Map sharpening *B* factor (Å^2^) | -500 | -150 | *N/A* |
| Model composition  Non-hydrogen atoms  Protein residues | 5695  750 | 5671  746 | *N/A*  *N/A* |
| *B* factors (Å^2^)  Protein | 164.03 | 413.26 | *N/A* |
| R.M.S. deviations  Bond lengths (Å)  Bond angles (°) | 0.003  0.726 | 0.002  0.681 | *N/A*  *N/A* |
| Validation  MolProbity score  Clashscore  Poor rotamers (%) | 2.23  18.29  0.00 | 2.27  18.83  0.48 | *N/A*  *N/A*  *N/A* |
| Ramachandran plot  Favored (%)  Allowed (%)  Disallowed (%) | 92.58  7.42  0.00 | 91.73  8.27  0.00 | *N/A*  *N/A*  *N/A* |

*N/A* = Not Applicable

**
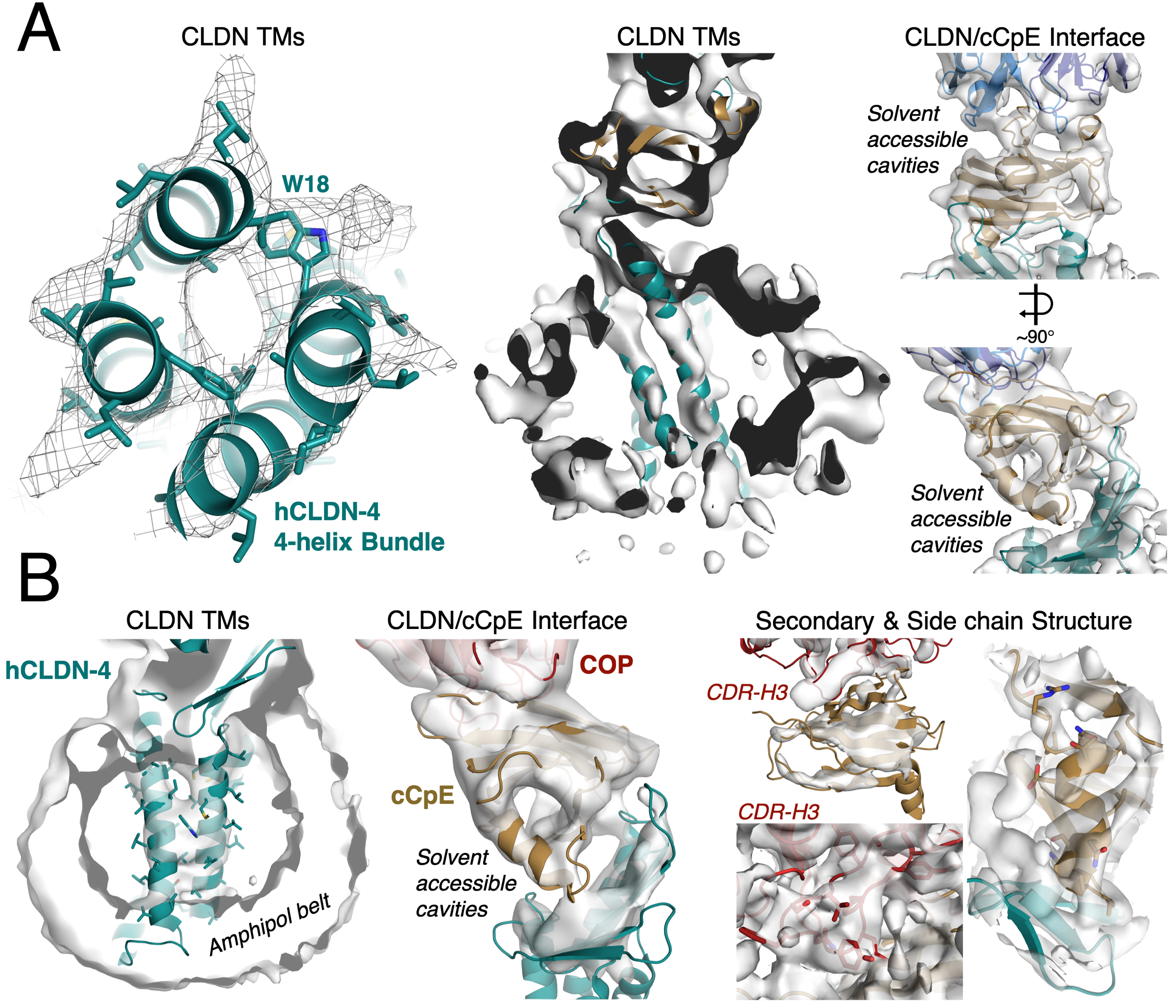
Figure S4. Cryo-EM map and structural features.** (A) Structure and corresponding 6.5 Å map from the human claudin-4 (hCLDN-4)/cCpE/COP-2 complex. Images depict: looking up through the TM bundle to the extracellular space with strong side chain density for Trp18 (left); a side view parallel to the membrane plane of individual TMs within the LMNG micelle (middle); and the claudin-4/cCpE and cCpE/COP-2 interfaces with various solvent accessible cavities resolved (right). (B) Structure and corresponding 4-4.5 Å map from the claudin-4/cCpE/COP-3 complex. Images depict: a side view parallel to the membrane plane of the TMs within the amphipol belt (left); claudin-4/cCpE interfaces with various solvent accessible cavities resolved (middle); and strand, helix, loop, and side chain density for aromatic amino acids at claudin-4/cCpE and cCpE/COP-3 interfaces (right). Proteins are shown as cartoons and colored as in Figs. 2 and 3.

**
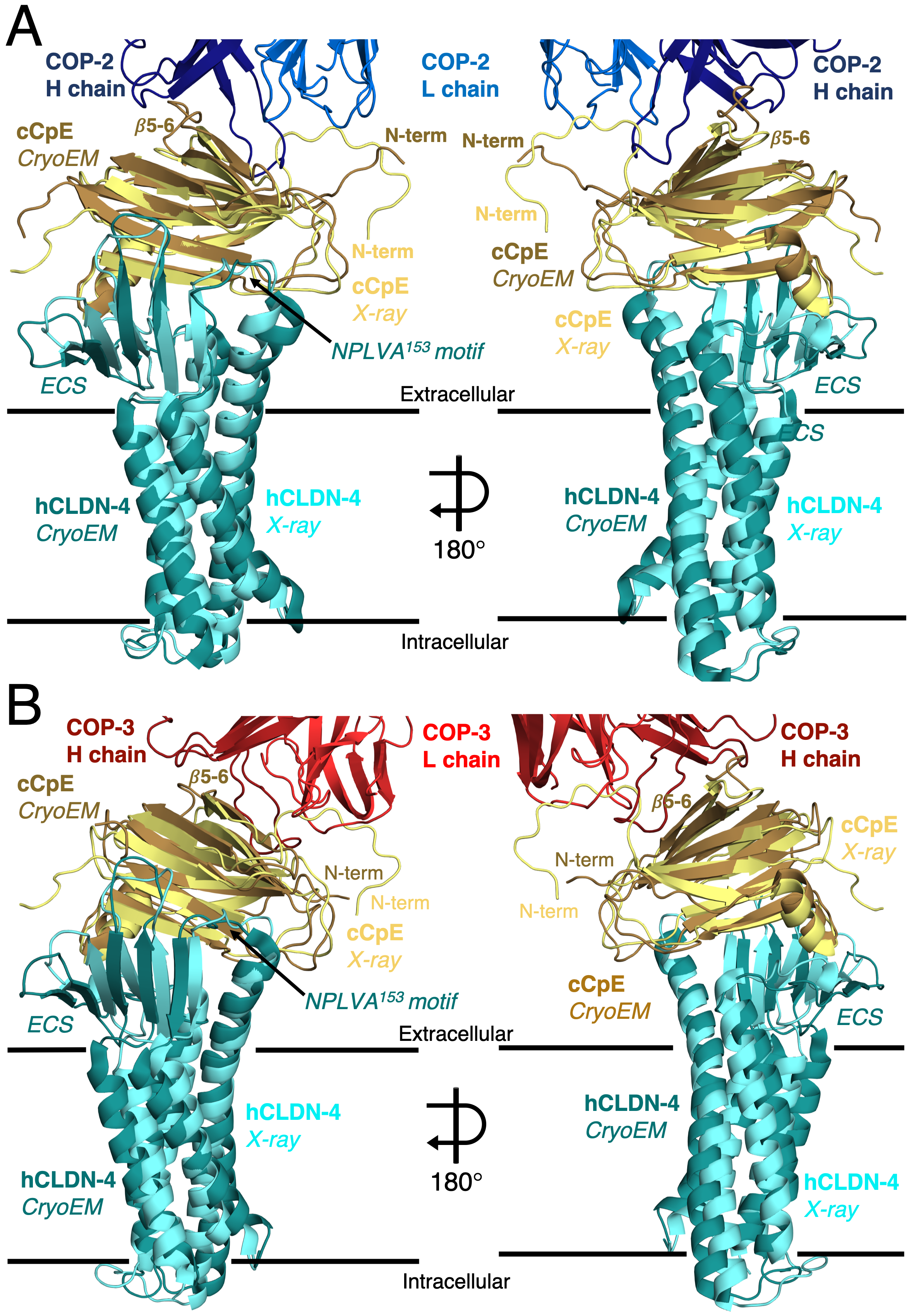
**

**Figure S5. Comparison of claudin-4/cCpE cryo-EM and crystal structures.** Structural overlays depicting similarity between human claudin-4 (hCLDN-4, teal)/cCpE (sand) complexes from cryo-EM, and claudin-4/cCpE (cyan/yellow) complex from X-ray crystal structure PDB ID 7KP4. (A) Overlay from COP-2-bound (blue) claudin-4/cCpE complex. (B) Overlay from COP-3-bound (red) claudin-4/cCpE complex. Proteins are shown as cartoons with model membrane borders shown as black lines.

**
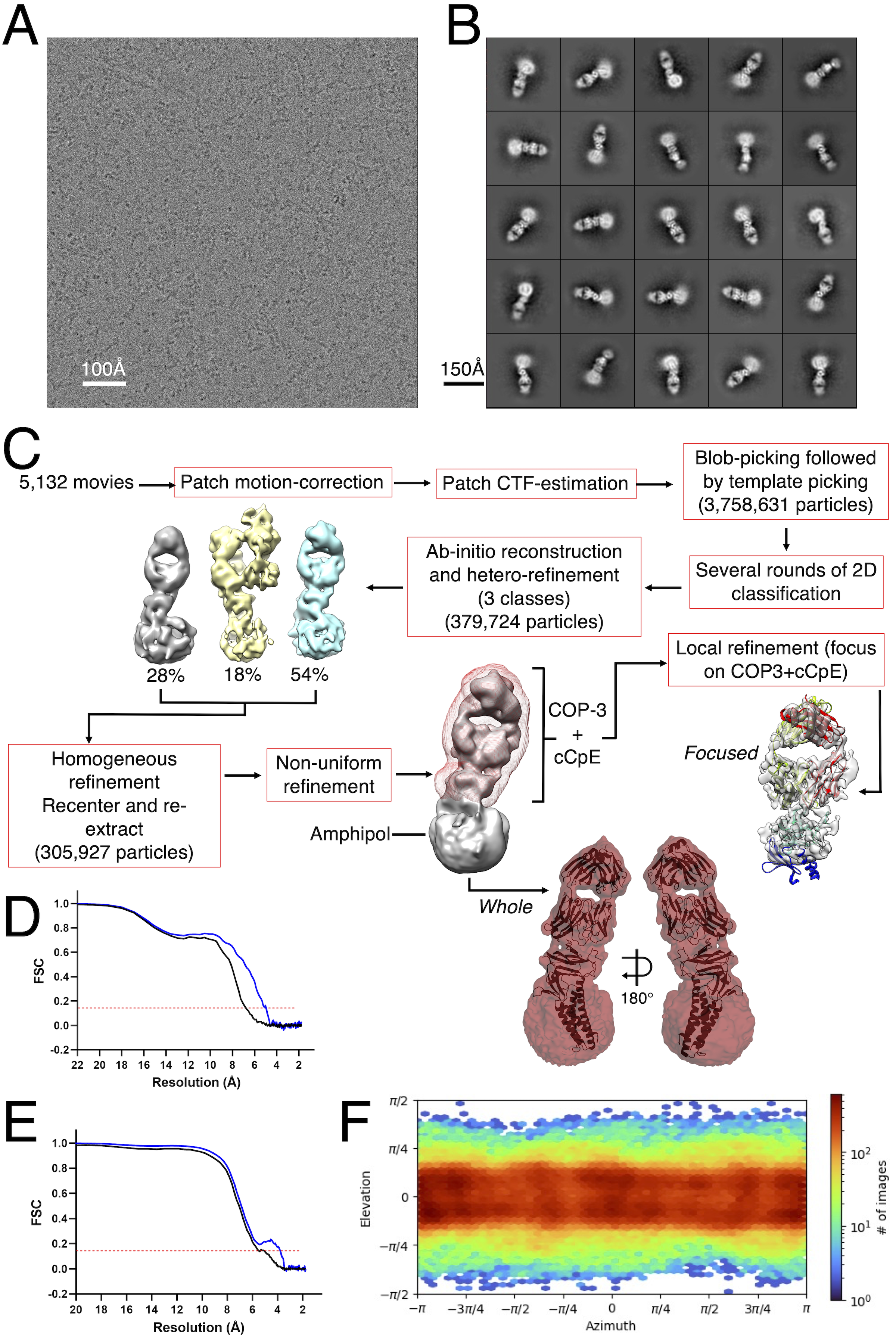
**

**Figure S6. Cryo-EM data processing for claudin-4/cCpE/COP-3 complex.** (A) Electron micrograph showing the distribution of claudin-4/cCpE/COP-3 complexes in ice. (B) Representative 2D class averages of claudin-4/cCpE/COP-3 complexes showing the clear binding of the COP-3 sFab, and signal for TMs within the amphipol belt. (C) Cryo-EM data processing workflow. (D) Fourier Shell Correlation (FSC) curves from gold-standard refinement of the entire complex. The 0.143 FSC cutoff is indicated by a dashed red line. The black line indicates FSC without a mask applied, and the blue line indicates FSC with a tight mask applied around the protein and amphipol belt. (E) Fourier Shell Correlation (FSC) curves from local refinement of the cCpE/COP-3 region. The 0.143 FSC cutoff is indicated by a dashed red line. The black line indicates FSC without a mask applied, and the blue line indicates FSC with a tight mask applied around the cCpE/COP-3 complex. (F) Angular distribution of particles in the final refinement.

**
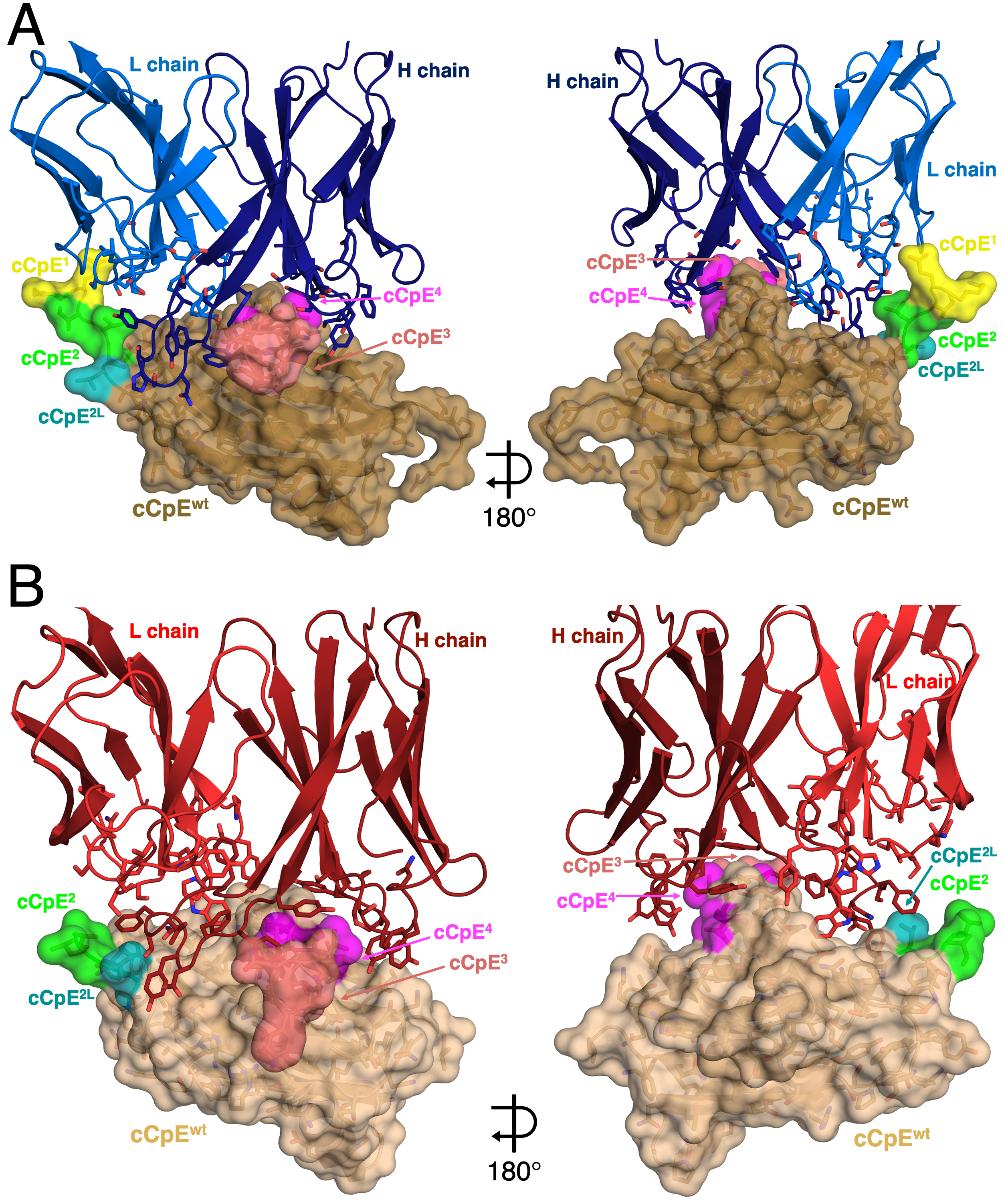
Figure S7. Modelled changes to COP surface epitopes for cCpE^mutants^.** (A) COP-2 (blue) bound to cCpE^wildtype^ (copper) with areas of cCpE^mutants^ colored as follows: mutant cCpE^1^ (yellow), cCpE^2^ (green), cCpE^2K^ (dark green), cCpE^2L^ (teal), cCpE^3^ (salmon), and cCpE^4^ (magenta). (B) COP-3 (red) bound to cCpE^wildtype^ (tan) with areas of cCpE^mutants^ colored as in A. COPs are shown as cartoons while cCpEs are shown as surfaces.

**
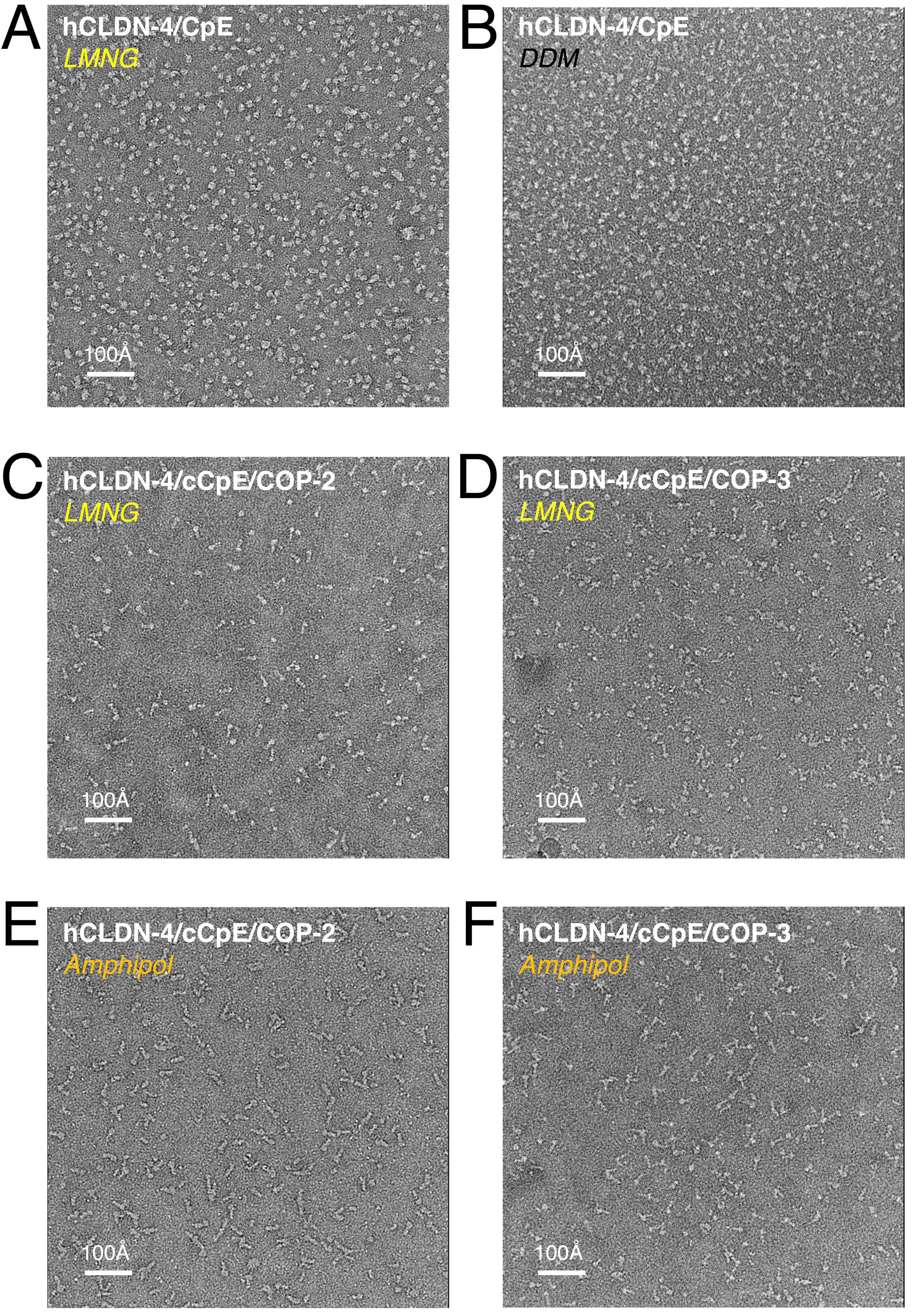
**

**Figure S8. Negative stain EM of claudin-4/enterotoxin complexes in various mimetics.** (A) Human claudin-4 (hCLDN-4) solubilized in LMNG bound to CpE. (B) hCLDN-4solubilized in DDM bound to CpE. (C) hCLDN-4 solubilized in LMNG bound to cCpE and COP-2. (D) hCLDN-4 solubilized in LMNG bound to cCpE and COP-3. (E) hCLDN-4 solubilized in amphipol bound to cCpE and COP-2. (F) hCLDN-4 solubilized in amphipol bound to cCpE and COP-3. All negative stain data were collected at 200 keV using a screening cryo-EM microscope. Samples from panels C and F were collected and further processed, resulting in the structures presented here.
